# Toward High Precision XCO_2_ Retrievals From TanSat Observations: Retrieval Improvement and Validation Against TCCON Measurements

**DOI:** 10.1029/2020JD032794

**Published:** 2020-11-09

**Authors:** D. Yang, H. Boesch, Y. Liu, P. Somkuti, Z. Cai, X. Chen, A. Di Noia, C. Lin, N. Lu, D. Lyu, R. J. Parker, L. Tian, M. Wang, A. Webb, L. Yao, Z. Yin, Y. Zheng, N. M. Deutscher, D. W. T. Griffith, F. Hase, R. Kivi, I. Morino, J. Notholt, H. Ohyama, D. F. Pollard, K. Shiomi, R. Sussmann, Y. Té, V. A. Velazco, T. Warneke, D. Wunch

**Affiliations:** ^1^ Earth Observation Science, School of Physics and Astronomy University of Leicester UK; ^2^ Institute of Atmospheric Physics Chinese Academy of Sciences China; ^3^ Shanghai Advanced Research Institute Chinese Academy of Sciences Shanghai China; ^4^ National Centre for Earth Observation University of Leicester UK; ^5^ Colorado State University Fort Collins CO USA; ^6^ Changchun Institute of Optics, Fine Mechanics and Physics China; ^7^ National Satellite Meteorological Center, China Meteorological Administration China; ^8^ Shanghai Engineering Center for Microsatellites China; ^9^ Centre for Atmospheric Chemistry, School of Earth, Atmospheric and Life Sciences University of Wollongong NSW Australia; ^10^ Karlsruhe Institute of Technology, IMK‐IFU Garmisch‐Partenkirchen Germany; ^11^ Space and Earth Observation Centre Finnish Meteorological Institute Finland; ^12^ National Institute for Environmental Studies (NIES) Tsukuba Ibaraki Japan; ^13^ Institute of Environmental Physics (IUP) University of Bremen Bremen Germany; ^14^ National Institute of Water and Atmospheric Research Ltd (NIWA) Lauder New Zealand; ^15^ Japan Aerospace Exploration Agency Japan; ^16^ Laboratoire d'Etudes du Rayonnement et de la Matière en Astrophysique et Atmosphères (LERMA‐IPSL) Sorbonne Université, CNRS, Observatoire de Paris, PSL Université Paris France; ^17^ University of Toronto Canada

**Keywords:** TanSat, CO2, satellite, retrieval algorithm

## Abstract

TanSat is the 1st Chinese carbon dioxide (CO_2_) measurement satellite, launched in 2016. In this study, the University of Leicester Full Physics (UoL‐FP) algorithm is implemented for TanSat nadir mode XCO_2_ retrievals. We develop a spectrum correction method to reduce the retrieval errors by the online fitting of an 8^th^ order Fourier series. The spectrum‐correction model and its a priori parameters are developed by analyzing the solar calibration measurement. This correction provides a significant improvement to the O_2_ A band retrieval. Accordingly, we extend the previous TanSat single CO_2_ weak band retrieval to a combined O_2_ A and CO_2_ weak band retrieval. A Genetic Algorithm (GA) has been applied to determine the threshold values of post‐screening filters. In total, 18.3% of the retrieved data is identified as high quality compared to the original measurements. The same quality control parameters have been used in a footprint independent multiple linear regression bias correction due to the strong correlation with the XCO_2_ retrieval error. Twenty sites of the Total Column Carbon Observing Network (TCCON) have been selected to validate our new approach for the TanSat XCO_2_ retrieval. We show that our new approach produces a significant improvement on the XCO_2_ retrieval accuracy and precision when compared to TCCON with an average bias and RMSE of −0.08 ppm and 1.47 ppm, respectively. The methods used in this study can help to improve the XCO_2_ retrieval from TanSat and subsequently the Level‐2 data production, and hence will be applied in the TanSat operational XCO_2_ processing.

## Key Points

- First using O_2_ A and 1.61 um CO_2_ band approaching TanSat XCO_2_ retreival
- Development a method on radiometric correction of TanSat L1B data in O_2_ A and 1.61 um CO_2_
- Validation of new TanSat retrieval against TCCON and recived significant improved results compare to previously retrieval

## 1 Introduction

Carbon Dioxide (CO_2_) has been recognized as the most important greenhouse gases causing climate change due to the rise in anthropogenic emissions since the industrial revolution. Accurate measurement of atmospheric CO_2_ in order to reduce the uncertainties of CO_2_ fluxes is a key requirement for meeting the “measurable, reportable and verifiable” mitigation commitments of the United Nations Framework Convention on Climate Change (UNFCCC) (https://unfccc.int/resource/docs/2007/cop13/eng/06a01.pdf) that is aimed at avoiding disastrous consequences caused by climate change. The existing in‐situ surface CO_2_ measurement networks provide a wealth of accurate data related to the global carbon cycle. Unfortunately, the sparse coverage of such networks is still a major limitation for global carbon cycle research and large uncertainties in our quantitative understanding of regional carbon fluxes remain.

The new generation of satellites with state‐of‐the‐art near infrared (NIR) and shortwave infrared (SWIR) hyperspectral spectrometers bring us a step closer toward global mapping of CO_2_ with sufficient accuracy, precision and coverage for reliable flux estimates on regional scales. Specifically, NIR/SWIR spectroscopy provides a means for measurements of the total column‐averaged dry air CO_2_ mole fraction (XCO_2_) which captures the CO_2_ signals in the lower troposphere including the boundary layer that can then be used to improve our knowledge on CO_2_ surface fluxes (Kuang et al., 2002).

The European Space Agency (ESA) SCanning Imaging Absorption SpectroMeter for Atmospheric CHartographY (SCIAMACHY) (Bovensmann et al., 1999) on board the ENVIronmental SATellite (ENVISAT) that launched in 2002 and operated until 2012, was the first space‐borne instrument to provide SWIR hyperspectral measurements of CO_2_ (Bösch et al., 2006; Buchwitz et al., 2005; Heymann et al., 2015; Reuter et al., 2011), followed by the Greenhouse Gases Observing Satellite (GOSAT) from Japan (Kuze et al., 2009) and the Orbiting Carbon Observatory‐2 (OCO‐2) from the U.S. (Crisp et al., 2008), launched in 2009 and 2014, respectively. These missions have significantly contributed to the effort to obtain global CO_2_ measurements from space (Eldering, O’Dell, et al., 2017; Yokota et al., 2009) and subsequently for carbon flux studies (Eldering, Wennberg, et al., 2017; Feng et al., 2017; Hakkarainen et al., 2016, 2019; Maksyutov et al., 2013; Yang et al., 2017). When validated against measurements from the Total Carbon Column Observing Network (TCCON) (Wunch, Toon, et al., 2011), XCO_2_ derived from GOSAT and OCO‐2 has an accuracy of better than 2 part per million (ppm) (Butz et al., 2011; Buchwitz, Dils, et al., 2017; Cogan et al., 2012; Crisp et al., 2012; Kim et al., 2016; O’Dell et al., 2012; Oshchepkov et al., 2013; Wu et al., 2018; Yang et al., 2015; Yoshida et al., 2013), thanks to the high performance of these instruments and long‐term calibration efforts (Crisp et al., 2017; Frankenberg et al., 2015; Kuze et al., 2014; Rosenberg et al., 2016; Yoshida et al., 2013). Further advances are expected from recently launched missions such as GOSAT‐2 (Nakajima et al., 2019) and OCO‐3 (Eldering et al., 2019) launched in 2018 and 2019 respectively, and future missions including MicroCarb (Bertaux et al., 2020) and CO2M (Kuhlmann et al., 2019).

China plays an important role in the global carbon budget as a major source of anthropogenic carbon (Le Quéré et al., 2018) due to its rapid economic development but also as a region of increased carbon sequestration thanks to a number of reforestation projects (Chen et al., 2019). In China, a series of ambitious projects on mitigation of carbon emission was kicked‐off in the last 10 years, which include the first Chinese greenhouse gas monitoring satellite mission (TanSat), which is supported by the Ministry of Science and Technology of China, the Chinese Academy of Sciences, and the China Meteorological Administration. The TanSat mission was initiated in 2011 and successfully launched on 22 Dec 2016. TanSat started acquiring and archiving data operationally in March 2017 (Chen et al., 2012; Ran & Li, 2019).

TanSat carries two instruments on‐board: the Atmospheric Carbon Dioxide Grating Spectrometer (ACGS) and the Cloud and Aerosol Polarimetry Imager (CAPI). ACGS is a state‐of‐the‐art hyperspectral grating spectrometer aimed at allowing XCO_2_ retrievals (Lin et al., 2017; Liu et al., 2013; Wang et al., 2014) by measuring backscattered sunlight in three NIR/SWIR bands: the oxygen (O_2_) A‐band (758–778 nm with ~0.04 nm spectral resolution), the CO_2_ weak band (1,594–1,624 nm with ~0.125 nm spectral resolution) and the CO_2_ strong band (2042–2082 nm with ~0.16 nm spectral resolution). CO_2_ column information is largely drawn from the weak CO_2_ band which includes a series of strong but not saturated CO_2_ lines which respond to even small variations in atmospheric CO_2_ (Bösch et al., 2006; Kuang et al., 2002). The O_2_ A band hyperspectral measurement contains information on aerosol and cloud scattering both in total scattering amount and scattering vertical distribution (Corradini & Cervino, 2006; Geddes & Bösch, 2015; Heidinger & Stephens, 2000). The strong CO_2_ band provides information on aerosol and cloud scattering at longer wavelengths, in conjunction with information on CO_2_ and water vapor (Wu et al., 2019). The CAPI is a multi‐band imager which provides radiance measurements in five bands (365‐408 nm, 660‐685 nm, 862‐877 nm, 1,360‐1,390 nm, 1,628‐1,654 nm) from UV to NIR. In order to achieve more information on aerosol size, which has a significant impact on the wavelength dependence of aerosol optical properties, CAPI includes two polarization channels (660‐685 nm and 1,628‐1,654 nm) to measure the Stokes parameters (Chen, Yang, et al., 2017).

TanSat flies in a sun‐synchronous low Earth orbit (LEO) with an equator crossing time around 13:30 local time. The satellite operates in three measurement modes: nadir (ND), glint (GL) and target (TG). GL provides routine measurements over oceans which are obtained by tracking the principle plane (the principal plane is spanned by the vectors from the sun to the surface footprint and from the surface point to the observer). ND provides the routine measurements over land with the satellite flying in consistent rotation angle routine as in GL mode. We only use ND observations in this study. The swath width of TanSat measurements is ~18 km across the satellite track and contains 9 footprints each with a size of 2 km × 2 km in nadir (Lin et al., 2017; Zhang et al., 2019). Nadir and glint mode alternate orbit‐by‐orbit, and the TanSat nadir model ground track is typically between two OCO‐2 tracks, which provides potential future opportunities for combined usage of both data products for increased spatial coverage.

The first global XCO_2_ dataset from TanSat observations for the first half year of operations has been produced with the Institute of Atmospheric Physics Carbon dioxide retrieval Algorithm for Satellite measurement (IAPCAS) (Yang et al., 2018), using the CO_2_ weak band only. The nadir XCO_2_ dataset has been evaluated using eight TCCON sites to show an average precision of 2.11 ppm for the TanSat retrievals (Liu et al., 2018).

In this study, we apply the University of Leicester Full Physics (UoL‐FP) algorithm to the TanSat XCO_2_ retrieval using a joint CO_2_ weak band and O_2_ A band retrieval, after adopting a new approach to radiometrically correct TanSat spectra. This new radiometric correction is introduced in Section 2 together with the UoL‐FP TanSat retrieval approach. The applied quality filtering and bias correction methods are discussed in Section 3. Section 4 gives the results of the comparisons of the TanSat XCO_2_ retrievals to ground‐based observations from the TCCON network and Section 5 provides the summary and outlook.

## 2 UoL‐FP TanSat XCO_2_ Retrieval Description

### 2.1 Introduction of the UoL‐FP Algorithm

The UoL‐FP is an XCO_2_ retrieval algorithm based on the Optimal Estimation Method (OEM) that has been originally developed for the NASA Orbiting Carbon Observatory (OCO) mission (Bösch et al., 2006), and has been extensively used for XCO_2_ retrievals from GOSAT (Cogan et al., 2012). Besides XCO_2_, the UoL‐FP has been used to retrieve methane (CH_4_) (Parker et al., 2011, 2015), HDO and H_2_O (Boesch et al., 2013) and Solar Induced chlorophyll Fluorescence (Somkuti et al., 2020). UoL‐FP is also one of the algorithms used to generate the Essential Climate Variables (ECV) XCO_2_ and XCH_4_ from GOSAT for the ESA Climate Change Initiative (CCI) (Buchwitz, Dils, et al., 2014 2017;) and subsequent European Commission Copernicus Climate Change Service (C3S) (Buchwitz, Reuter, et al., 2017).

Several publications have already introduced the UoL‐FP algorithm and its applications in detail (Boesch et al., 2013; Cogan et al., 2012; Parker et al., 2011) and here we only provide a brief overview. Basically, the retrieval aims to resolve an optimization problem to find the best estimate of a state vector $\hat{x}$ by minimizing the difference between a measured and a simulated spectrum taking into consideration additional constraints on the measurement errors and state vector a priori uncertainties. This state vector gives all retrieved parameters and includes atmospheric, surface and instrument variables. Full physics refers to the fact that the algorithm generates the simulated spectrum via an accurate multiple‐scattering Radiative Transfer (RT) model. As many processes involved in the transfer of light through the atmosphere respond in a non‐linear way to changes in state vector elements, an iterative Levenberg–Marquardt (L‐M) inversion scheme is used in the retrieval,

(1) $$x_{i+1}=x_{i}+\left[\left(1+\lambda \right)S_{a}^{-1}+K_{i}^{T}S_{\varepsilon }^{-1}K_{i}\right)]{}^{-1}[K_{i}^{T}S_{\varepsilon }^{-1}\left(y-F\left(x_{i}\right))-S_{a}^{-1}\left(x_{i}-x_{a}\right)\right],$$

where ***x***_***a***_ is the a priori of the state vector. ***S***_***a***_ and ***S***_***ε***_ indicate the covariances of the state vector and the measurement respectively. The weighting function (known as Jacobians) ***K*** gives the linear change of the spectrum per change in state vector *∂****y***/*∂****x***. The update step of the state vector’s *i* ^th^ iteration from ***x***_*i*_ to ***x***_*i*+1_ can be adjusted by the L‐M factor *λ*.

The forward model, which includes a vector RT model, is one of the most essential parts of the retrieval algorithm. In UoL‐FP, the radiative transfer model LIDORT is used, which is a linearized discrete ordinate radiative transfer model that generates radiances and Jacobians simultaneously (Spurr et al., 2001; Spurr & Christi, 2014). According to the instrument design, ACGS/TanSat only measures one direction of polarized light instead of the total intensity; hence we need to compute the Stokes vector {*I*, *Q, U, V*} (Liou, 2002; Mishchenko et al., 2004; Stokes, 1852) in the forward simulation. Since multiple scattering is depolarizing, it is reasonable to expect that the polarization could be accounted for by a low‐order scattering approximation. For a relatively clear atmosphere (e.g. aerosol optical depth <0.3), retaining only the second order of scattering components for *Q* and *U* is generally sufficient (Natraj & Spurr, 2007). A 2‐orders of scattering (2OS) model (Natraj & Spurr, 2007) is applied in our RT model to extend the scalar LIDORT to vector simulation capability (Somkuti, 2018; Somkuti et al., 2017). The Low Stream Interpolation (LSI) method is used to speed‐up the RT calculations (O’Dell, 2010).

The atmosphere is discretized into 20 coarse layers from the surface up to 0.1 hPa allowing 10 sublayers within each coarse layer (200 fine layers in total) to reduce interpolation errors from non‐linear changes of the gas absorption cross sections with pressure and temperature. Absorption cross sections for O_2_, CO_2_, H_2_O and CH_4_ are considered in the RT computation. We use the NASA ACOS/OCO‐2 v5.0 absorption coefficients (ABSCO) that have been extensively used in ACOS GOSAT and OCO‐2 retrievals (Benner et al., 2016; Devi et al., 2016; Drouin et al., 2017).

UoL‐FP employs two aerosol retrieval types representing large and small aerosol particles. The optical depth profile and optical properties for each type are inferred from the aerosol data from the Copernicus Atmosphere Monitoring Service (CAMS) (https://atmosphere.copernicus.eu/). There are five basic aerosol types, including sea salt, dust, organic matter, black carbon and sulphate, provided by CAMS. Beyond the type, we also consider hydrophobic and hydrophilic effects in computing optical properties, as organic matter and black carbon are separated into hydrophobic and hydrophilic particles, whereas sea salt and sulphate are treated as hydrophilic, and dust as hydrophobic only. For aerosol concentration and vertical distribution, we use the CAMS model data as a climatology which is created from the CAMS data for the years 2014–2016. In addition, a high‐altitude cirrus retrieval type is included using the ice particle model of Baum et al. (2014). The surface reflectance is assumed to be Lambertian and is described by a mean albedo and its wavelength dependent slope for each band.

### 2.2 Polarization

As shown by the sensitivity study of Natraja et al. (2007) and Bai et al. (2018), significant errors could be introduced in the O_2_ A band and CO_2_ weak band RT computation of radiances when using intensity instead of a combination of Stokes components, which can then cause significant errors in the CO_2_ retrieval due to the wrong surface pressure and aerosol contributions (Butz et al., 2009; Geddes & Bösch, 2015).

In physical terms, the light measured by the instrument can be represented by a linear combination of Stokes components {*I*,*Q*,*U*,*V*},

(2) $$L=a_{1}I+a_{2}Q+a_{3}U+a_{4}V,$$

As the circular polarization component *V* is very weak in a realistic atmosphere, we ignore this parameter by setting *a*_4_ to 0. The Stokes parameter coefficients *a*_1_, *a*_2_ and *a*_3_ are determined by the satellite position, measurement geometry and the pointing direction of the polarizer, which are provided in the L1B data product. More detail on the definition of the polarization angle is given in Appendix A.

### 2.3 Radiometric Correction Approach of TanSat Spectra

#### 2.3.1 TanSat Solar Calibration Measurement

TanSat has multiple on‐orbit calibration strategies, including solar, dark field and white light lamp calibration, which are helpful to monitor the instrument status and performance. TanSat switches to solar calibration measurements when it flies over the ascending end of each orbit until almost in darkness and regularly takes ~7 minutes (~1,260 frames) of solar measurements. The solar calibration procedure provides direct measurements of the solar spectrum which has no contamination from the Earth’s atmosphere and surface and thus without uncertainties from the radiative transfer of light. During the on‐orbit test phase, solar calibration is performed once every two orbits, and then changes to daily during operational observations.

Ideally, we can imagine the solar calibration as a model for a measurement essentially without atmospheric extinction and scattering (there is a well‐calibrated solar diffuser used in the solar calibration, which involves a reflection). Therefore, one can use solar calibration measurements to verify the radiometric and spectral calibration applied to a measured spectrum if an accurate solar model is used and the reflection from the diffuser is well known (Wang et al., 2018).

Here, we use the new solar line model (2016 version) created by G. C. Toon (2014) (https://mark4sun.jpl.nasa.gov/toon/solar/solar_spectrum.html) combined with the UoL‐FP solar continuum model that is obtained from a polynomial fitting of SOLar SPECtrometer (SOLSPEC) measurements (Meftah et al., 2018). The solar line model has been extensively used and verified with GOSAT and OCO‐2 retrievals (Uchino et al., 2012). This solar model combines the solar lines and solar continuum, and hence can be directly used for solar model fitting without any further calculation.

TanSat observes the Sun though a reflective diffuser before the relay optical system, hence the wavelength‐dependent diffuser reflectance needs to be compensated for, otherwise it could cause an extra pattern in the measured solar spectrum. In this study, we use a wavelength‐dependent Bidirectional Reflectance Distribution Function (BRDF) that has been characterized pre‐flight in the laboratory (Wang et al., 2014) without any corrections for a time dependent degradation as the instrument performance is stable. The solar irradiance is non‐polarized and we use a factor of 0.5 to adjust for the linear polarizer.

To avoid contamination of terrestrial absorption from the upper atmosphere when the satellite observes the limb measurement region, only measurements with boresight solar zenith angle (defined angle between line of sight and solar) between 5° and 6° are used in fitting. Note that TanSat rotates by a 5° pitch angle (away from the Earth) to avoid damage of CAPI from strong incident light (Chen, Yang, et al., 2017; Chen, Wang, et al., 2017).

#### 2.3.2 Solar Calibration Analysis and Radiometric Corrections

**Figure 1:**
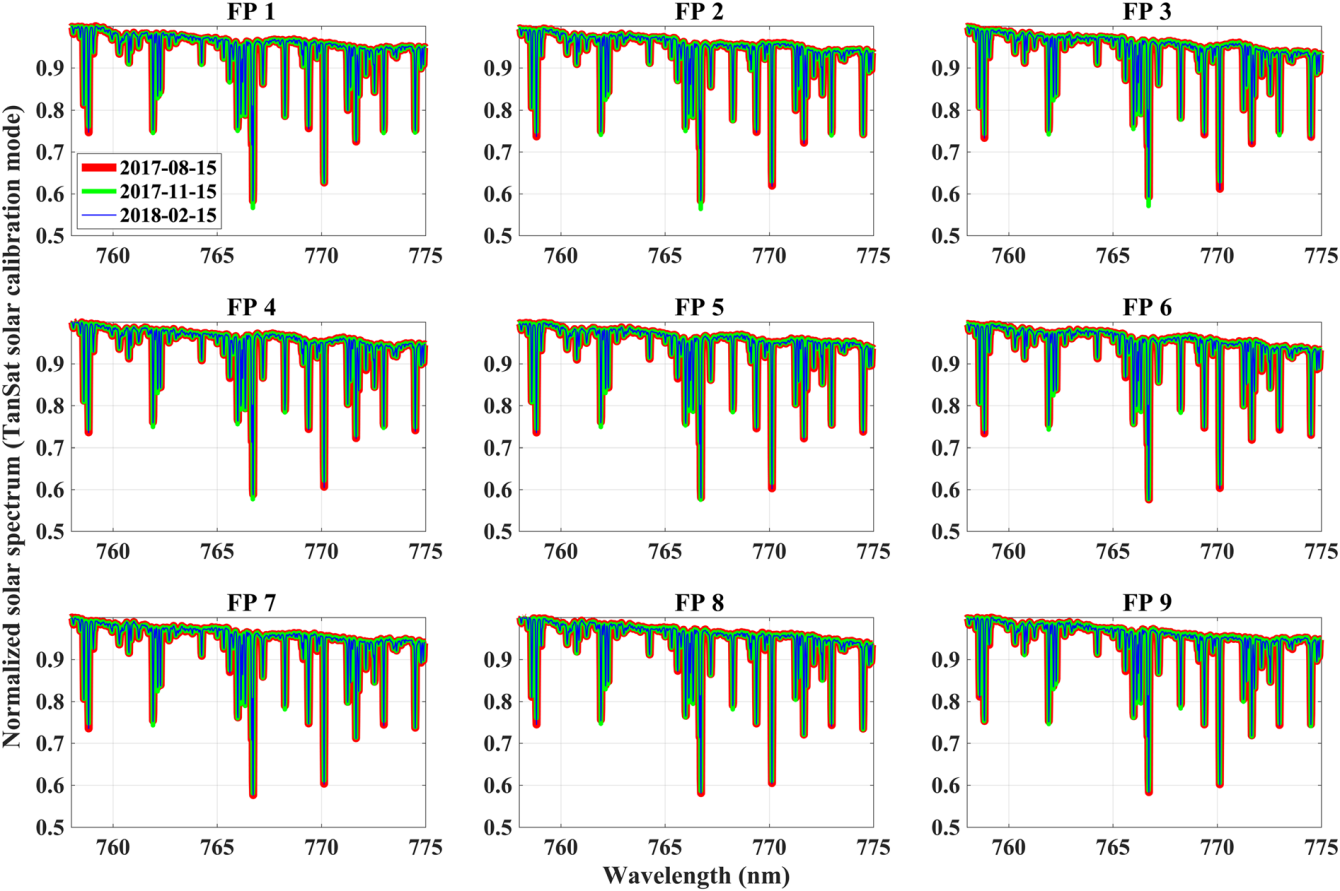
Mean spectrum of the normalized solar calibration measurement of the O_2_ A band. The average is calculated for each calibration measurement in an orbit. Red, green and blue indicate the mean measurement taken for 2017/08/15, 2017/11/15 and 2018/02/15 respectively. Sub‐figures represent the footprints (FP1–9) across the swath. The date is shown in the legend of FP1 sub‐figure.

Monitoring the solar calibration spectrum shows a stable instrument performance during the first year of TanSat operations (Figure 1, the CO_2_ weak band is also stable but this is not shown here). The mean normalized solar measurement in the 750 nm region shows little change (mean normalized spectrum changes < 0.1%) for spectra acquired every 3 months, except for a very small difference for solar lines, which is probably caused by small instrument performance changes, e.g. instrument line shape (ILS). The figure shows that spectra acquired for the different cross‐track footprints show some differences to each other in the continuum, which means each footprint has to be analyzed separately.

In order to further investigate the solar calibration spectrum, we developed a fitting tool for the solar spectrum based on the solar model corrected for the variable Earth‐Sun distance and the Doppler shift effect (in wavelength) due to Earth’s rotation and revolution. The fitting tool retrieves the wavelength dependence continuum correction in different forms (e.g. polynomial or Fourier series) as well as a wavelength shift and stretch simultaneously. The fitting tool uses the Gaussian‐Newton method without the constraint of measurement noise.

**Figure 2:**
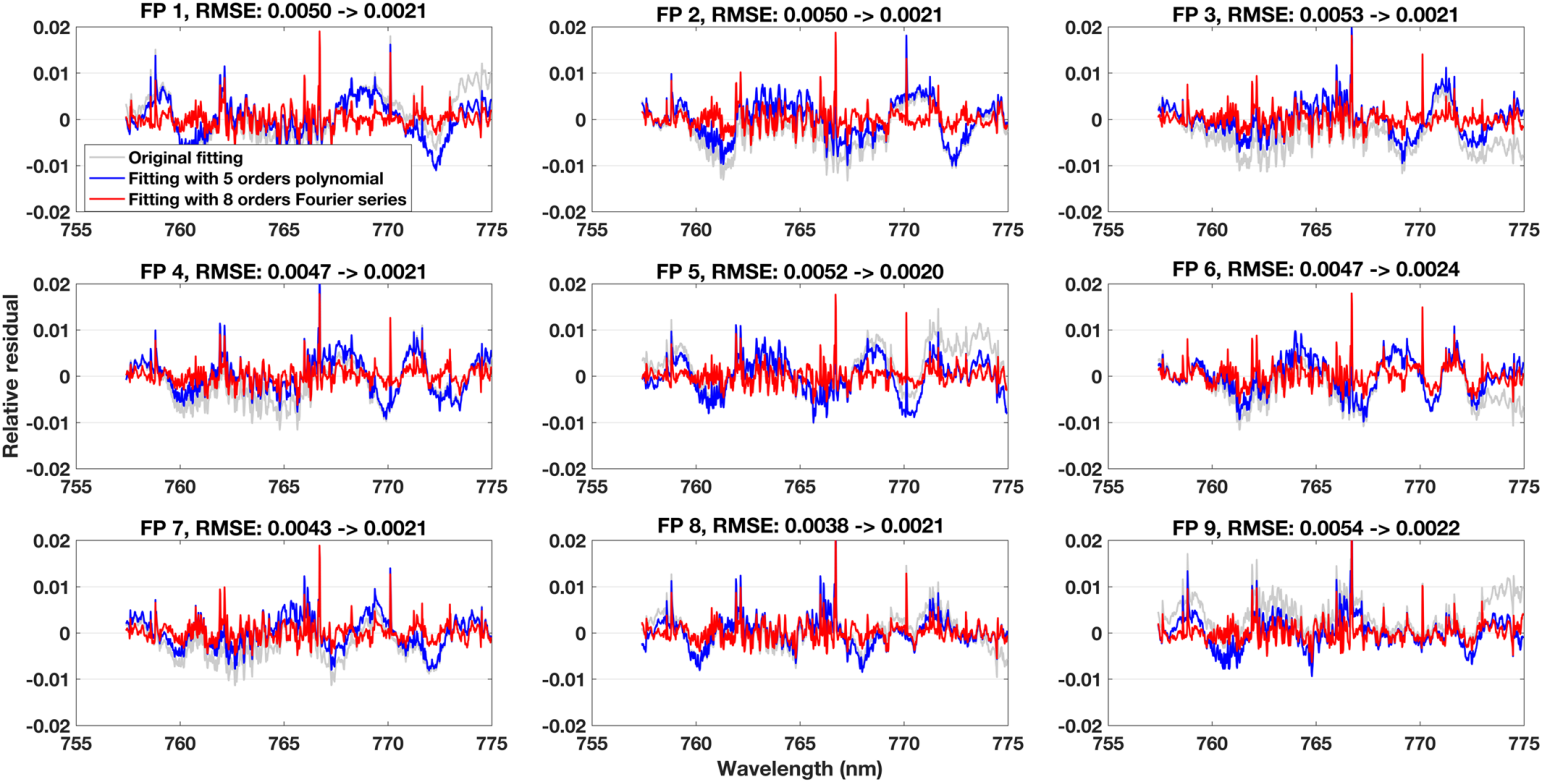
Mean fitting residuals of the solar calibration measurements. The average is calculated from the fitting residuals of 181,232 individual spectra (for each footprint, the quantity is ~20,136) during mar. 2017 – May 2018. The blue and red lines show the mean residual obtained by fitting a 5^th^ order polynomials or a 8^th^ order Fourier series model, respectively (see the detail in text section 2.3). The light gray line is the fitting residual when fitting a scale factor without any wavelength dependent corrections. Sub‐figures indicate the 9 footprints (FP1‐FP9) across the swath. The RMSE shown in the title of each sub‐figure shows the improvement of the Fourier series model compared to using the wavelength‐independent scale factor.

The fitting algorithm is fast and hence we fit all individual solar spectra between March 2017 and May 2018 without averaging or manual spectrum selection. In total, 181,232 retrievals have been carried out and we find consistent fitting residuals for each cross‐track footprint, which is expected considering the stable solar calibration measurement (Figure 1). Averaged relative fitting residuals for the NIR band are given in Figure 2. A considerable and consistent pattern with a mean RMSE of 0.48% remained in the fitting residual when we only adjust a wavelength‐independent scaling factor to the continuum, which needs to be corrected to avoid errors in the XCO_2_ retrieval. Thus, a method is needed that can compensate for these structures and improve the fitting residual. However, this pattern is not a simple linear or quadratic function with wavelength and a more complex model is needed. We found that the relative residual did not change much with changes in intensity of the incident light introduced by instrument degradation and Earth‐Sun distance changes and hence we adopt a correction model based on multiplicative continuum scaling rather than a model using additive offsets.

A common approach is to use a polynomial as a function of wavelength to scale the continuum (*L*_*inc*_). We have found that a 5^th^ order polynomial is the best choice,

(3) $$L=\left(\sum_{i=0}^{5}a_{i}\cdot {\Delta \lambda }^{i}\right)\cdot L_{\mathrm{inc}}+D\left(\lambda \right),$$

where *a*_*i*_ is the coefficient of the *i* ^*t*h^ order of the polynomial component for a wavelength relative (Δ*λ* = *λ* − *λ*_0_, *λ*_0_ is the reference wavelength, in this study, we use first wavelength grid point of each band) to a reference of 750 nm. *D*(*λ*) represents an additive offset assumed to be a linear function given by a slope and a constant. This additive offset could compensate the impact from SIF (only O_2_ A band) and stray light. As can be seen in Figure 2, this polynomial approach leads only to minor improvements in the fitting residual. Increasing the order of the polynomial did not lead to significant improvements compared to the 5^th^ order.

An alternative approach that should have a better capability to capture the oscillating nature of the fitting residuals is to use a Fourier series:

(4) $$L=\left(c+\sum_{i=1}^{n}\left(a_{i}\cdot \cos\left(i\cdot \omega \cdot \Delta \lambda \right)+b_{i}\cdot \sin\left(i\cdot \omega \cdot \Delta \lambda \right)\right)\right)\cdot L_{\mathrm{inc}}+D\left(\lambda \right),$$

where *a*_*i*_ and *b*_*i*_ are coefficients of *i* ^th^ order of the Fourier series cosine and sine components with *c* being the zero‐order coefficient, and *ω* the scaling coefficient for frequency. Δ*λ* and *D*(*λ*) are the same as for the polynomial model above. We have tested a 5^th^ to 10^th^ order Fourier series in the solar calibration fitting and finally found that a 8^th^ order is the best choice, which is because less than 8^th^ order is not sufficiently compensating the fitting residual and more than 8 leads to limited further improvement compared to 8^th^ orders. As a result of using Fourier series model, we find a significant improvement in the fitting residual with a mean RMSE of 0.21% (Figure 2) compared to 0.48% for the polynomial approach. Note that, (1) the fitting residual near solar lines is still larger than the measurement noise, and (2) a gas absorption structure is visible in the residuals, which is also the case in fit residuals for the CO_2_ weak band. This could be correlated with stray light from Earth or preflight calibration (e.g. ILS and radiometric calibration), but the exact reason is unclear and needs to be further investigated in the future. In principal, the continuum correction corrects the dominant effects in the fitting residual but some features still remain visible in the residuals. For example, larger residual structures appear for solar lines. Considering that this affects only a small number of pixels throughout a spectral band, it can be expected that impact on the retrieval is limited and we do not attempt to further correct these features. This continuum feature is probably caused by several reasons, e.g. radiometric calibration and stray light. For nadir observation, the component of incident light is more complicated than for solar calibration due to the scattering and absorption in atmosphere. However, we found similar residual features in both non‐corrected solar calibaration and nadir observation fitting. Therefore, the applied Fourier series is applicable to nadir observation. But the parameters change between solar calibration and nadir observation fitting and also among the soundings, and hence we retrieve all Fourier series parameters in the cloud screening and XCO_2_ retrieval (See details in Text S3, Table S2 and Figure S6, S7of SI).

The applied continuum correction in our study is basically a correction of the radiometric gain and no other corrections are applied. This is because (1) errors of the continuum are more obvious and they are stable in the case of TanSat, (2) solar lines are not sufficiently deep to constrain potential non‐linearity corrections, (3) the ILS cannot be easily re‐analyzed from space‐based data, especially since TanSat did not provide solar calibration measurement for the entire dayside which would scan the ILS due to the Doppler shift (Sun et al., 2017).

### 2.4 O_2_ A Band Surface Pressure Retrieval for Cloud Screening

The O_2_ A band is important in the XCO_2_ retrieval because, (1) it allows cloud screening based on apparent surface pressure to remove measurements that are highly contaminated by thick cloud, and (2) to provide information on aerosol and thin clouds in a joint O_2_ A and CO_2_ band retrieval to reduce errors that are otherwise introduced by light path modification from scattering.

An Oxygen A‐Band (ABO2) cloud‐screening algorithm is used as the cloud screening algorithm based on the analysis of a small number of micro‐windows in the O_2_ A band. The ABO2 algorithm has been applied to GOSAT and OCO‐2 cloud screening and verified against MODIS and CALIOP measurement (Taylor et al., 2012, 2016). Unfortunately, the continuum correction described above cannot easily be applied to a retrieval that uses narrow micro‐windows, and benefit from the fast RT model. Hence, we adopt an apparent surface pressure retrieval (assuming aerosol‐free conditions) based on a fast RT model for the whole range of the O_2_ A band (0.757–0.772 μm) that covers a multitude of O_2_ lines and continuum for both sides of the band. This retrieval includes surface pressure, temperature profile offset, Lambertian surface albedo and its wavelength dependence slope, wavelength stretch and the coefficients of the Fourier series continuum model in the NIR as retrieved parameters. The outputs are used later as a priori values for a subsequent XCO_2_ retrieval (Table 1). A priori surface pressure is taken from the European Centre for Medium‐Range Weather Forecasts (ECMWF) ERA‐Interim 0.75° × 0.75° reanalysis data product (Dee et al., 2011), and is interpolated to the location of the sounding and corrected for elevation differences using the U.S. Geological Survey’s (USGS) EROS Data Center Shuttle Radar Topography Mission Global 30 Arc‐Second Elevation (SRTM30) digital elevation model (DEM) (ftp://edcsgs9.cr.usgs.gov/pub/data/srtm/SRTM30).

**Table 1:** List of state vector elements and descriptions for the UoL‐FP/TanSat retrieval

Acronym	Description	N	A priori	A priori error (1σ)
CO_2_ profile	Carbon dioxide (CO_2_) concentration on each layer surface	21	LMDZ MACC‐II CO_2_ model	
H2O scale	Scaling for water vapor (H_2_O) profile	1	ECMWF interim 0.75°	$\sqrt[{2}]{0.1}$
Temperature shift	Shift for temperature profile	1	ECMWF interim 0.75°	$\sqrt[{2}]{10}$ K
P surf	Surface pressure	1	ECMWF interim 0.75°	2 hPa
Albedo B1	Surface albedo of oxygen (O_2_) A band	1	Estimate from spectrum continuum level	1
Albedo B1S	Surface albedo wavelength dependence slop of O_2_ A band	1	0	0.0042
Albedo B2	Surface albedo of CO_2_ weak band	1	Estimate from spectrum continuum level	1
Albedo B2S	Surface albedo wavelength dependence slope of CO_2_ weak band	1	0	0.01
Aerosol M1	The profile of the most dominant aerosol type	21	Copernicus Atmosphere Monitoring Service (CAMS)	
Aerosol M2	The profile of the 2^nd^ dominant aerosol type	21	Copernicus Atmosphere Monitoring Service (CAMS)	
Cirrus	The profile of cirrus	21		
Zeroff B1	The zero offset of O_2_ A band	1	0	1% of continuum (SIF signal)
Zeroff B1S	The zero offset wavelength dependence slope of O_2_ A band	1	0	1
Zeroff B2	The zero offset of CO_2_ weak band	1	0	10% of continuum level
Zeroff B2S	The zero offset wavelength dependence slope of CO_2_ weak band	1	0	1
Continuum B1	Fourier series correction coefficients on continuum of O_2_ A band, 1 frequency scale and 16 coefficients of trigonometric function	17	Fitting results from long term solar calibration measurement	Comes from experimental value
Continuum B2	Fourier series correction coefficients on continuum of CO_2_ band, 1 frequency scale and 16 coefficients of trigonometric function	17	Fitting results from long term solar calibration measurement	Comes from experimental value
Dispersion B1	Polynomials on wavelength grid for dispersion of O_2_ A band	6	L1B data, dispersion coefficients O_2_ A	Fixed with experimental value
Dispersion B2	Polynomials on wavelength grid for dispersion of CO_2_ weak band	6	L1B data, dispersion coefficients CO_2_ weak	Fixed with experimental value

We found that the frequency coefficient (***ω*** in equation (8)) of the Fourier series cannot be well‐fitted in the O_2_ surface pressure retrieval if the first guess is not very close to the true value due to non‐linear effects. Since we observe that the structure in the fitting residuals of the solar spectra changes little with time we can obtain a good first guess from the fitting of solar calibration measurement value, not only for ***ω*** but also for other coefficients. A set of 20,000 high quality solar calibration soundings (RMSE < 0.21%, which is the mean RMSE of all retrievals) including all 9 footprints have been selected for this calculation.

Another highly non‐linear parameter is the stretch of the wavelength grid. The update of TanSat L1B data from version 1 to version 2 significantly improved the wavelength calibration, but additional corrections are still necessary. The solar calibration fitting also simultaneously provides wavelength stretch coefficients which we then use in the O_2_ A band surface pressure retrieval.

**Figure 3:**
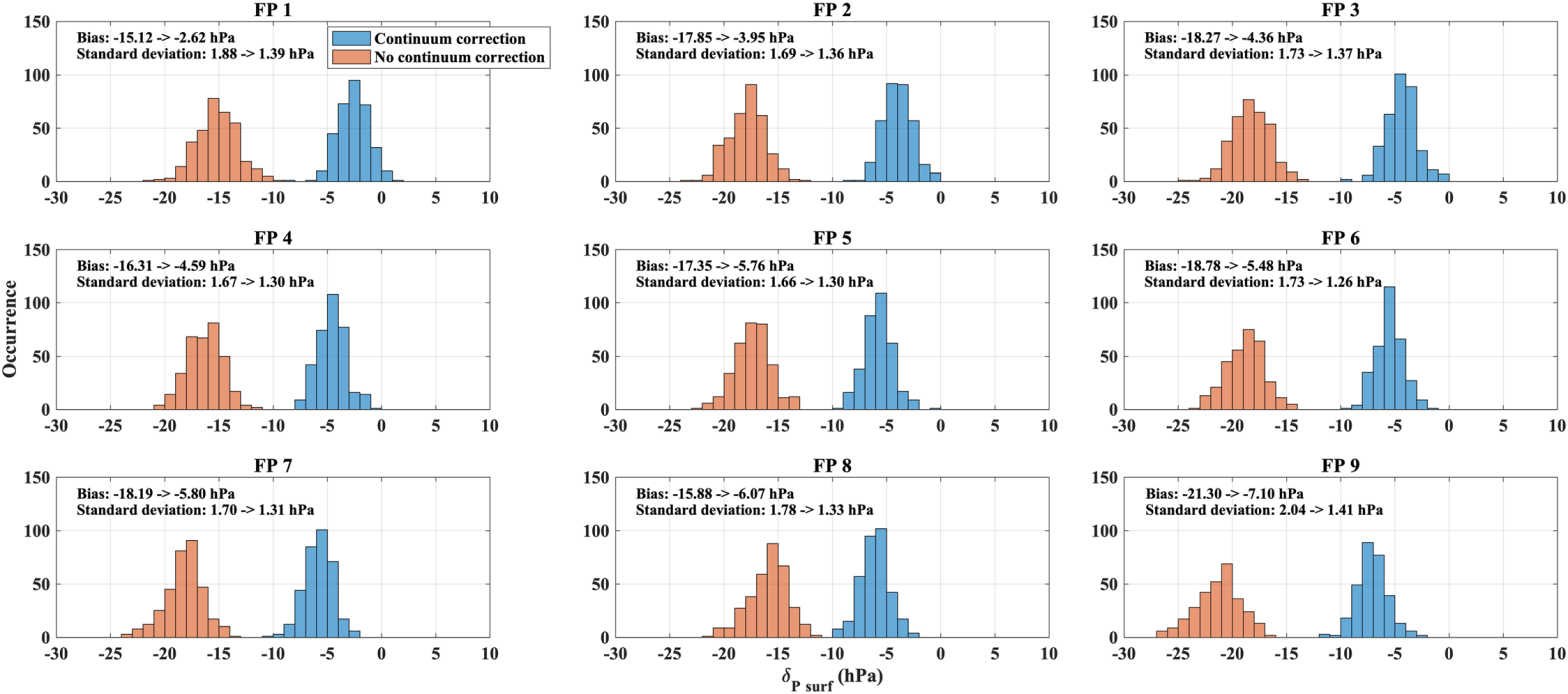
Histograms of surface pressure changes (*δ*_*P surf*_) from O_2_ A band retrievals with (blue) and without (orange) Fourier series continuum correction for selected clear‐sky conditions (selected cases on 2017‐10‐08) overpass TCCON/Lamont (USA) site. The changes are calculated by subtracting the a priori (height corrected surface pressure from ECMWF interim) from the retrieved apparent surface pressure. The sub‐figures show the footprints (FP1‐FP9) across the swath. The retrieved apparent surface pressure with continuum correction still shows a small bias to the a priori, because the retrieval has been carried out without any consideration of aerosol and cloud scattering (see the detail in text section 2.4).

The impact of the Fourier continuum correction on the surface pressure retrieval is significant (Figure 3). We found that (1) the retrieval without the correction has a large bias and scatter, and (2) there are large differences between the cross‐track footprints in the retrieval even after continuum correction (with a constant gain factor). Therefore, the application of the apparent surface pressure retrieval for cloud screening without continuum correction is problematic due to the different scatter and bias for different footprints which could mean that a large number of clear‐sky measurement will be screened out for some footprints. As is shown for the case given in Figure 3, the surface pressure values for the retrieval with continuum correction mostly fall in the range between −10 to 0 hPa which satisfies the commonly used criterion for cloud screening of −20 to +20 hPa. In contrast, the retrieval without correction spreads between −10 to −30 hPa.

A ± 20 hPa threshold for cloud screening is reasonable and relatively loose, which means more data is allowed to pass the cloud screening. The benefit is obvious: we do not include aerosol and cloud scattering corrections in the apparent surface pressure retrieval, so that there will always be a small bias in the retrieved surface pressure which can become more significant for very dark surfaces (Figure 3).

### 2.5 TanSat XCO_2_ Two‐Band Retrieval

The information on CO_2_ volume mixing ratio (VMR) comes to a large extent from the 1.6 μm CO_2_ weak band, which means that a retrieval based on only the weak band can provide a relatively accurate result if the measurement scene is not impacted by aerosols or if perfect knowledge on aerosols is available. Unfortunately, both are not possible for real scenarios. The preliminary TanSat XCO_2_ retrieval (version 1.0) has been created using the CO_2_ weak band only (Liu et al., 2018; Yang et al., 2018). An extremely stringent filter has been applied in post screening, and hence screens out a large number of retrievals. The data produced with this approach provides good information on the global CO_2_ distribution and trend but does not yield enough quantity or accuracy for reliable surface flux inversions.

The O_2_ A band surface pressure retrieval with our newly developed continuum correction shows reliable results; hence we can extend the CO_2_ retrieval with confidence to use the O_2_ A and CO_2_ weak band together to improve the retrieval accuracy. We have applied a two‐band retrieval to produce XCO_2_ data from soundings that are recognized as clear‐sky scenes by the cloud screening. The retrieval uses a state vector that includes a CO_2_ profile, scale factors for temperature and water vapor, surface pressure, surface albedo and spectral slope. In addition, we fit an additive zero offset and its wavelength dependent slope in both the O_2_ A and CO_2_ weak bands. For the CO_2_ weak band, the same correction model as for the O_2_ A band is applied. The full state vector is given in Table 1.

**Figure 4:**
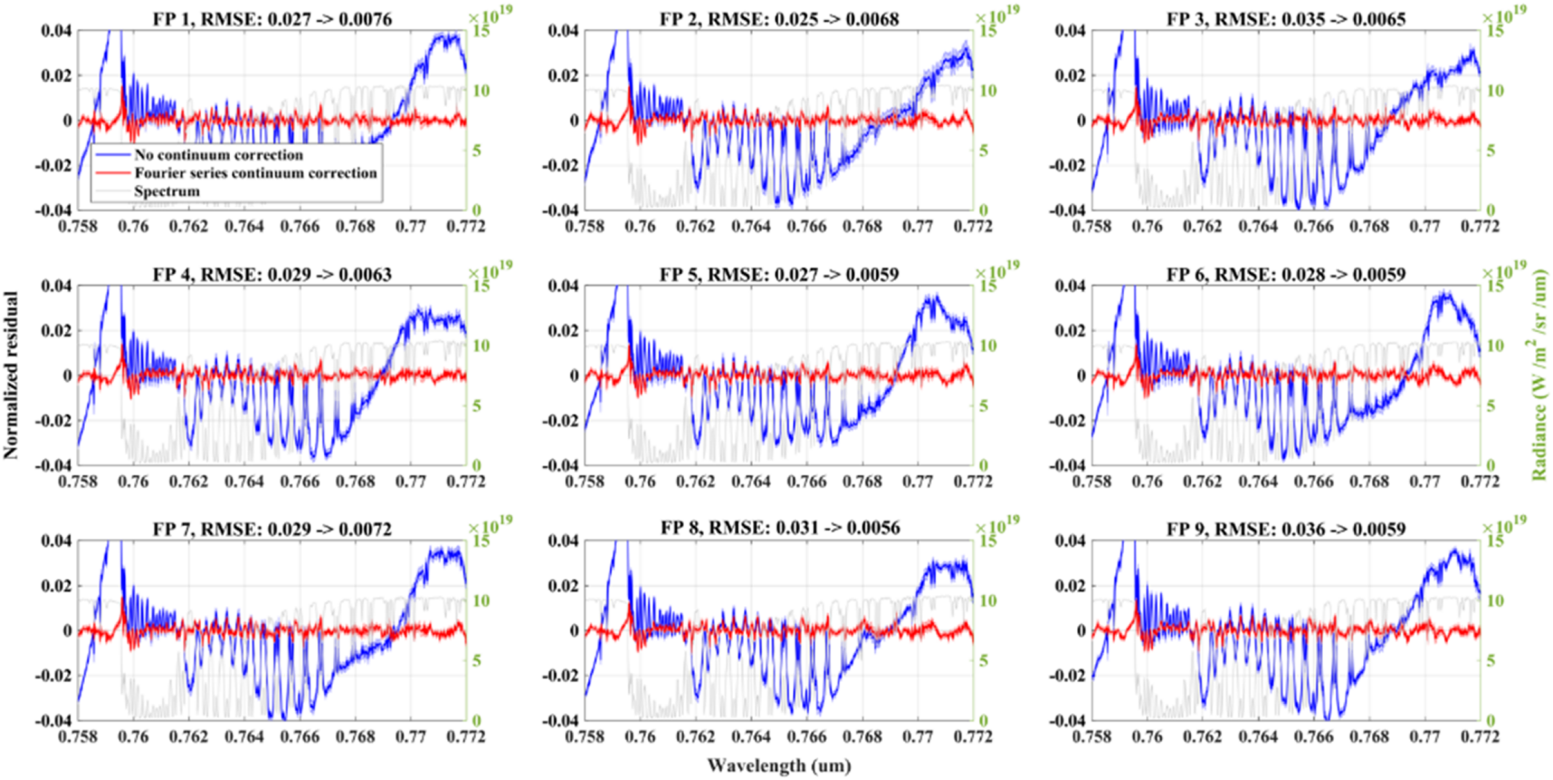
Mean O_2_ A band fitting residuals (normalized by continuum level) from the two bands retrieval with (red) and without (blue) Fourier series continuum correction. The average is computed from selected clear‐sky measurements on 08/10/2017 around the TCCON/Lamont site. The corresponding MODIS/aqua RGB image is shown in Figure 3. The light gray line (right y‐axis) shows the measurement spectrum as reference. The red and blue shading indicates the continuum normalized standard deviation (SD) for the retrieval with and without Fourier series continuum correction respectively.

**Figure 5:**
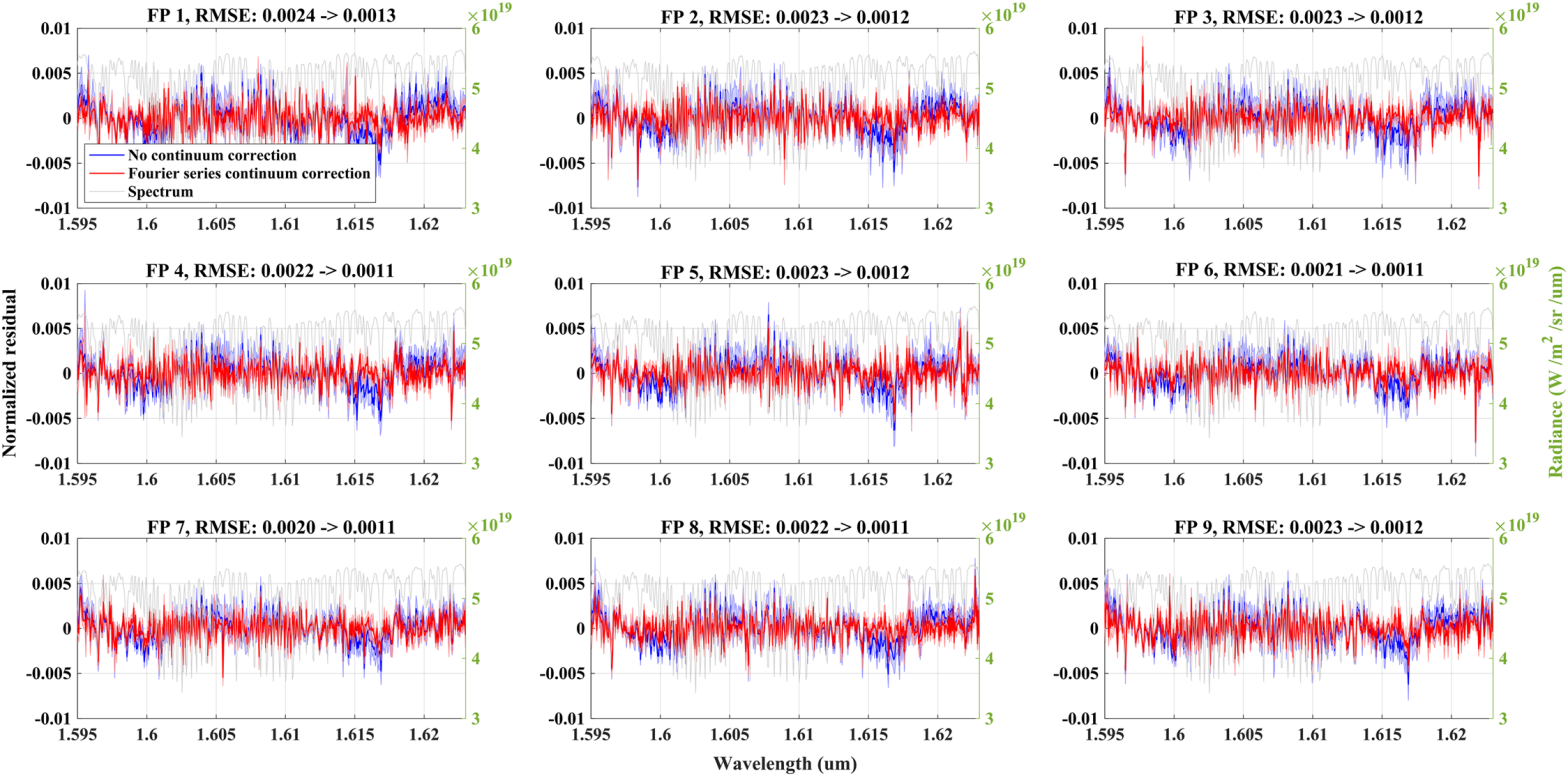
Same as Figure 4, but for the CO_2_ weak band.

Significant improvements to the O_2_ A band residual have been found when using the Fourier series correction. The Standard Deviation (SD) of the normalized residual indicates that the fitting is stable (Figure 4). Notice that the residuals of the 9 footprints show a slight difference even with the correction, e.g. at the long‐wavelength end that contain few atmospheric absorption features, which means this correction cannot perfectly compensate all of the spectral patterns. The residual still contains structures related to O_2_ lines, which is because (1) the spectroscopy is not perfect (Connor et al., 2016), and (2) the observed residual features for the solar calibration fitting which we discussed in section 2.3. The improvement for the CO_2_ weak band is also large (Figure 5) and the RMSE is reduced by almost half for the retrieval with the continuum correction. Similar to the O_2_ A band, minor residual patterns remain and need to be investigated in the future.

## 3 Quality Control and Bias Correction

### 3.1 Dataset

To remove outliers and to correct for small biases, a quality control filter and bias correction is applied using a reference dataset that is reliable enough for indicating retrieval errors. The Total Carbon Column Observing Network (TCCON) provides accurate measurement of XCO2 (Wunch, Toon, et al., 2011; Wunch et al., 2015), and has been used for GOSAT and OCO‐2 filtering and bias correction (Kim et al., 2016; Oshchepkov et al., 2013; Wunch, Wennberg, et al., 2011; Wunch et al., 2017; Wu et al., 2018; Yoshida et al., 2013). The OCO‐2 team has also developed additional methods for filtering and bias correction for their version 8 product, including small area approximation, multi‐model median and southern hemisphere approximation (O'Dell et al., 2018). In this study we only focus on the retrieval around TCCON sites, and hence only the data of 20 TCCON sites (Table 2) has been selected as a reference dataset for bias/filtering and validation in this study.

**Table 2:** The TCCON site list used in the validation study and the site validation statistics

Site	Date range		Validation	
		N	bias (ppm)	RMSE (ppm)
Bialystok, Poland (Deutscher et al., 2019)	20,090,301–20,180,427	2	0.78	0.93
Bremen, Germany (Notholt et al., 2019)	20,070,115–20,180,420	1	−0.29	0.29
Burgos, Philippines (Morino, Velazco, et al., 2018; Velazco et al., 2017)	20,170,303–20,180,427	2	0.27	1.10
Darwin, Australia (Griffith, Deutscher, et al., 2014)	20,050,828–20,180,308	12	0.29	1.36
East Trout Lake, Canada (Wunch et al., 2018)	20,161,007–20,181,231	19	0.21	1.12
Edwards, USA (Iraci et al., 2016)	20,130,720–20,181,231	3	1.36	1.39
Garmisch, Germany (Sussmann & Rettinger, 2018a)	20,070,716–20,181,220	5	0.24	1.18
JPL, USA (Wennberg et al., 2014)	20,110,519–20,180,514	20	−1.12	1.39
Karlsruhe, Germany. (Hase et al., 2015)	20,100,419–20,190,121	6	0.33	1.67
Lamont, USA (Wennberg et al., 2016)	20,080,706–20,181,231	17	0.37	0.76
Lauder, New Zealand (Sherlock et al., 2014)	20,100,202–20,181,031	9	1.19	1.40
Orléans, France (Warneke et al., 2019)	20,090,829–20,180,405	2	1.40	1.83
Paris, France. (Té et al., 2014)	20,140,923–20,180,126	4	0.048	0.62
Park Falls, USA (Wennberg et al., 2017)	20,040,602–20,181,229	15	0.41	1.20
Pasadena, USA (Wennberg et al., 2015)	20,120,920–20,181,231	19	−1.41	1.84
Rikubetsu, Japan (Morino, Yokozeki, et al., 2018)	20,131,116–20,180,423	4	−0.85	1.12
Sodankylä Finland (Kivi et al., 2014)	20,090,516–20,181,030	9	1.17 (0.35)^a^	2.83 (1.25) ^*^
Saga, Japan (Shiomi et al., 2014)	20,110,728–20,181,021	13	−0.92	1.53
Tsukuba, Japan (Morino, Matsuzaki, et al., 2018)	20,110,804–20,180,427	7	−1.04	1.62
Wollongong, Australia (Griffith, Velazco, et al., 2014)	20,080,626–20,180,425	5	0.90	1.23
Zugspitze, Germany (Sussmann & Rettinger, 2018b)	20,150,424–20,181,218	‐	‐	‐

^a^ Large bias point removed

The quality control (filtering), bias correction and validation is based on an inter‐comparison of UoL‐FP/TanSat retrievals against TCCON measurement, and those two results have been obtained from two different instruments and retrieval algorithms with different averaging kernels and a priori assumptions. A method for removing smoothing error differences caused by the different instruments and retrieval algorithm (Rodgers & Connor, 2003) has been used in GOSAT (Cogan et al., 2012) and OCO‐2 validation (O'Dell et al., 2018; Wunch et al., 2017). In practice, the application of this correction has led to small changes of <0.3 ppm (O'Dell et al., 2018; Nguyen et al., 2014). In this study, we directly compare the TanSat retrieval with TCCON measurement without attempting to remove differences in smoothing errors.

A colocation criterion of ±3° in latitude/longitude for matching up the TanSat soundings and TCCON measurements is applied. Other spatial co‐location methods exist which allow to increase the quantity of matched up data, which is important for GOSAT (Guerlet et al., 2013; Wunch, Wennberg, et al., 2011), but since TanSat has a much higher data density compared to GOSAT, the spatial criterion above does provide a sufficient number of soundings. All TCCON measurements within ±1 hour of the satellite overpass are averaged to provide a reference dataset for each overpass, and only overpasses with more than 20 TCCON measurements within the 2 hour period are used. In this study, we found 396,068 co‐located TanSat retrievals in 174 overpasses across the 20 TCCON sites.

Using the TCCON average as reference, we define the individual retrieval error as the XCO2 difference between each TanSat retrieval ( ${\hat{C}}_{\text{TanSat}}$) and the TCCON average ( ${\bar{C}}_{\text{TCCON}}$) for a satellite overpass: $\;\delta_{{\mathrm{xco}}_{2}}={\hat{C}}_{\text{TanSat}}-{\bar{C}}_{\text{TCCON}}$. Hence, there will be hundreds of individual TanSat retrievals for each average TCCON value for an overpass, except when heavily contaminated by clouds.

### 3.2 Semi‐Autonomous Sounding Selection

#### 3.2.1 Method

The retrieval algorithm adjusts a number of parameters related to the atmosphere, surface and instrument and due to the complexity and non‐linearity of the retrieval problem and limitations of the forward model to perfectly model the real behavior of the instrument, some retrievals will converge to a false solution or a local minimum. Therefore, as a first step, a quality control filter is applied to the retrievals to screen out such outliers before using the XCO_2_ retrievals for bias correction and validation.

The main goal of a filter design is to efficiently screen out the poor retrievals (defined by large individual errors $\delta_{{\mathrm{xco}}_{2}}$) while keeping a maximum number of high‐quality retrievals. Assuming that large errors are introduced by an imperfect forward model and/or measurement, we expect errors to correlate with other parameters used in the retrieval that are adjusted together with CO_2_.

The quality control normally comes with two basic questions, namely how to select the filter parameters and how to decide the best threshold values.

To answer the first question, we select 37 candidate filters and calculate the correlations with $\delta_{{\mathrm{xco}}_{2}}$ (only the top 8 have been listed in Table 3). The significance of each candidate filter is sorted with respect to the correlation coefficient, and the candidates with the lowest impact on $\delta_{{\mathrm{xco}}_{2}}$ (correlation coefficient < 0.3) are abandoned at the beginning. We select the candidates according to their rank, which means the first candidates have the highest priority to be chosen. Actually, it is not possible to decide on the best choice of filters (we call the number of filters complexity from hereon in) before the analysis on the performance for all possible complexity options has been carried out.

**Table 3:** Selected filter used in quality control and corresponding lower and upper thresholds

Name	Description	Lower boundary	Upper boundary
Grad CO2	The retrieval changes of layer CO_2_ gradient between 700 hPa and the surface	−4.34	21.47
Delta Psurf	The retrieval changes on surface pressure from a priori	−4.45	1.99
Continuum B1C3	Continuum correction coefficients of cos(x) of O_2_ A band	−0.76	0.60
Zeroff B2S	Zero offset wavelength dependence slope of CO_2_ band	−0.14	0.017
AlbedoB2	Surface albedo of CO_2_ weak band	0.033	0.33
Zeroff B1S	Zero offset wavelength dependence slope of O_2_ A band	‐	‐
H2Oscale	Scale factor of H_2_O	‐	‐
Continuum B1C4	Continuum correction coefficients of sin(x) of O_2_ A band	‐	‐

**Figure 6:**
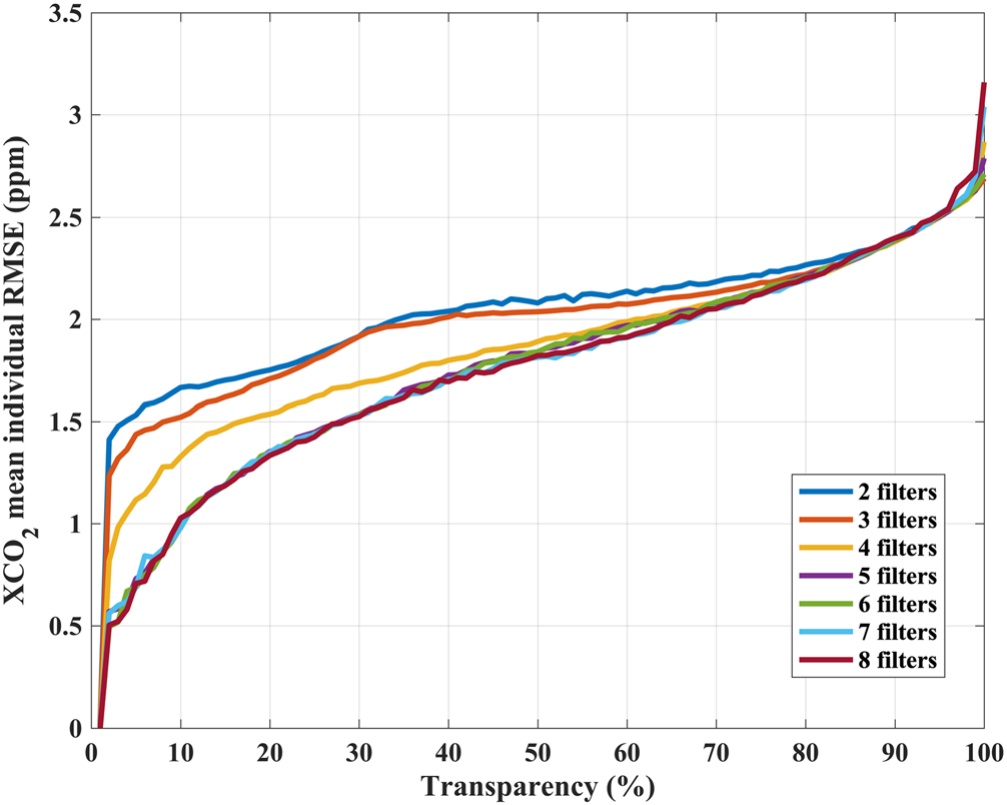
The optimal target run genetic algorithm (GA) profile (Pareto‐optimal trade‐off curves) for the selection of filter complexity and transparency. The XCO_2_ mean individual RMSE is a total RMSE calculated from all retrievals which pass the optimized threshold value (good retrieval). The color indicates the number of parameters (complexity) that are used in the GA run. For each complexity, the filter is determined and fixed (see section 3.2.1 for details and Table 3 for filter definitions). Transparency has a 1% interval through 0–100%. No significant additional reduction in the RMSE was seen when using more than 5 filters. In this study, we cut‐off at 2 ppm with 5 filters, which is an ad hoc choice, and the transparency is 64.3%. The 2 ppm RMSE is a compromise between coverage and accuracy. The RMSE is calculated from individual TCCON overpass couples, which means the footprint can be spatially away from the TCCON location, and hence there are would be geolocation differences that casue naturally CO_2_ differences. In addition, we also have to consider the measurement error and bias that include in the RMSE.

The solution to the latter question is often approached in an empirical manner. Physical basis methods have been developed and applied in the NASA Atmospheric CO_2_ Observations from Space (ACOS) OCO‐2 retrieval for versions V7 (Wunch, Toon, et al., 2011), and in subsequent versions V8 (O'Dell et al., 2018) and V9 (Kiel et al., 2019). In this study, we hope to use a method that is less driven by empirical decisions. A machine learning Genetic Algorithm (GA) method has been developed and applied to OCO‐2 to generate warn level data (Mandrake et al., 2013). The algorithm optimizes complexity (how many filters are selected), threshold value and transparency (how many data points pass the filter, represented by percentage) simultaneously. Mandrake et al. (2013) use one additional species of gene to control the filter selection and optimize this selection simultaneously, which treats each candidate equally, and then causes the filter combination for each complexity to be different. In this study, the filter has been selected according to the rank of the $\delta_{{\mathrm{xco}}_{2}}$ correlation, which means that the selection of the filter combination is carried out after the complexity is fixed. For each GA, runs with different complexity from 2 to 8 are carried out and shown in Figure 6. We optimize the threshold values of all filters for each GA run with chosen complexity with different transparency simultaneously (see more details of the GA that is used in this paper in Appendix B).

Subjective selection of transparency and complexity is required at the end of the applied GA. The transparency for different complexities against RMSE is shown in Figure 6. It needs to be noted that for each complexity the filter is fixed, which means the complexity +1 represents the addition of an extra filter (Table 3). Few improvements on filter selection have been found when the complexity is larger than 4 for the determination of OCO‐2 warn levels (Mandrake et al., 2013). We also found similar results for a complexity of 5 when compared to the complexity runs with values of 2–8 (Figure 6). The advantages of multi‐feature combinations for more than 4 features appears only when the fraction of filtered‐out data is close to 50%, and there are few advantages if the fraction of filtered‐out data becomes less than 30% (transparency > 70%). For a target of 2 ppm RMSE, we select a transparency between 64–65% (64.3%) with five filters.

#### 3.2.2 Application of the Filter

**Figure 7:**
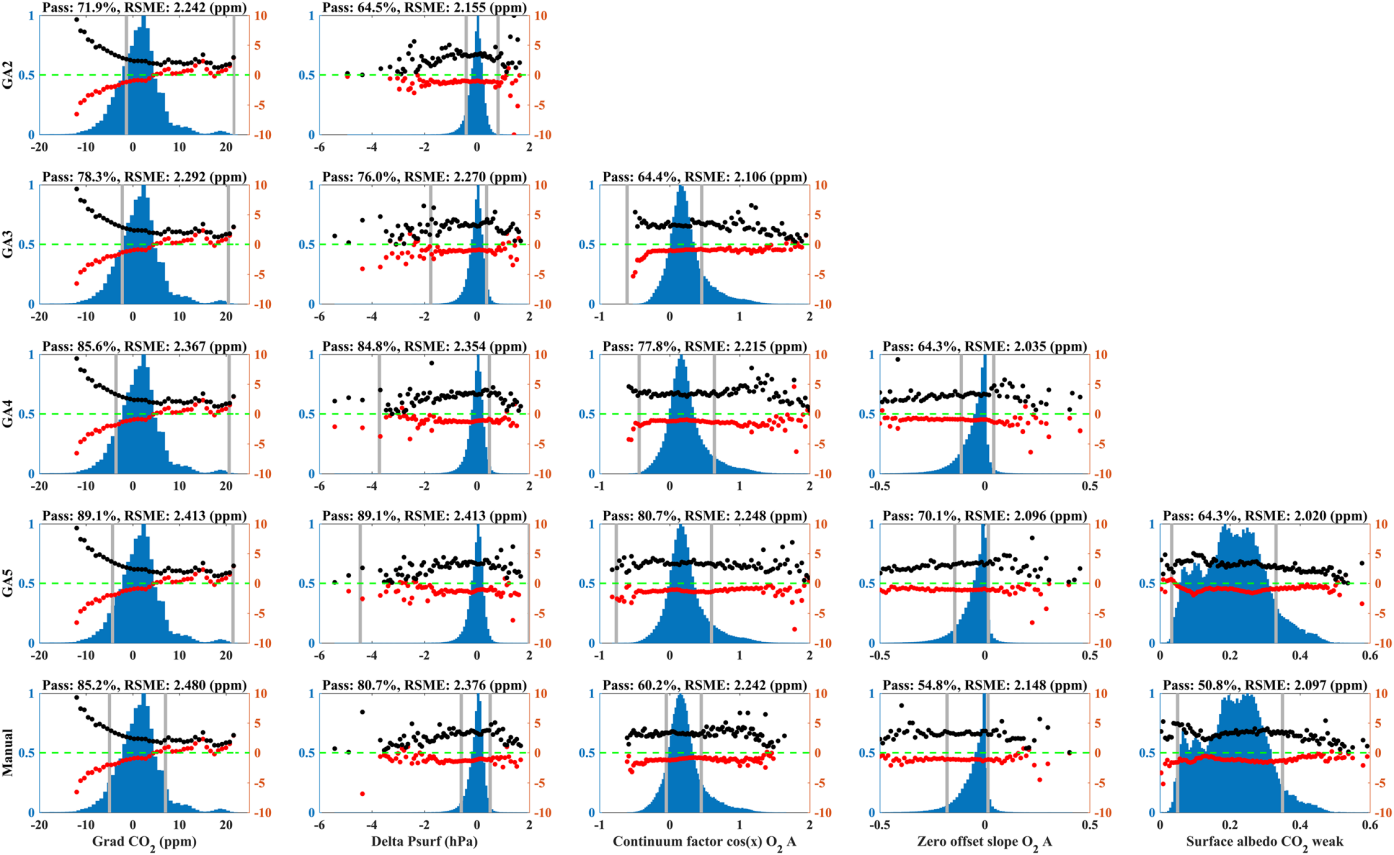
The performance of the GA with 2–5 filters (rows 1–4) and a manual filter threshold selection with 5 filters (row 5). The histograms (left y‐axis) indicate the counts for each filter bin, and red and black solid points show the bias and RMSE for each bin (right y‐axis). The gray line is the upper and lower threshold used for each filter. The filters are applied from left to right (columns 1–5) sequentially.

Retaining as much data as possible for a given requirement of RSME is an advantage of the GA method. However, GA can give an optimal solution in a mathematical sense, which may not be physically reasonable; namely the filter thresholds are unrealistic. For contrasting the results obtained with GA against manual selection, we also applied an empirical selection of filter thresholds for the five first filters applied in GA (Table 3). The results are compared in Figure 7 using the bin‐error plot method which is similar to that used by O’Dell et al. (2018) for OCO‐2 post screening. In general, we find that the thresholds from GA and the empirical method (manual selection) have similar effects on the filtering. However, to achieve a similar RSME with the empirical selection of thresholds, we reduce the amount filtered data by an additional 13.5% compared to GA.

Some filters, e.g. Grad CO_2_, Delta Psurf, and AlbedoB2, are parameters which have a specific physical meaning. Although these parameters are constrained during the retrieval, unreasonable results still occasionally occur. The GA that has been applied in this study has no capability of judging if a threshold has a reasonable value or not. Therefore, in practice, we strongly recommend applying additional physical filters to remove unreliable retrievals, if needed. However, in this study we only use GA screening.

In summary, our retrieval convergence rate is 94.8% of the cloud‐free measurement (~30% of the original measurements for a 20 hPa threshold of the apparent surface pressure from the O_2_ A band) and 64.3% have been recognized as good retrievals by GA. In total, we keep ~18.3% of all nadir measurements, and subsequently apply a bias correction to them.

### 3.3 Bias Correction

The bias correction is applied after the quality control. Biases in retrieved XCO_2_ can be introduced by shortcomings in the retrieval algorithm (e.g. parameterized models and radiative transfer), the measurements (e.g. stray light or calibration) and the databases which are used (e.g. gas absorption and solar model). The former two will typically lead to variable biases that will depend on other parameters while the latter one more likely induces a global bias. Commonly, the parameter‐dependent bias dominates and can be corrected (Wunch, Wennberg, et al., 2011) using a linear combination of identified bias parameters, $\Delta_{\mathrm{XCO}2}=\sum_{i=1}^{n}a_{i}\cdot p_{i}+b$, with *a*_*i*_ being the linear coefficient for the i^th^ parameter *p*_*i*_, and *b* an offset.

For the bias correction, we use the filters that have already been applied in the quality control as these five parameters are most strongly correlated with the error in XCO_2_. A multiple linear regression is applied to find the coefficients ***a***_***i***_ for each across‐track footprint. A further small improvement in RMSE is found with increasing the number of parameters up to 16 (SI section 1). The most significant improvement appears for the first three parameters and further parameters have a smaller effect. Improvement becomes less significant when using more than 12 parameters. Here, we use five parameters to avoid over‐optimizing the bias correction. The number and selection of filter parameters is somewhat subjective and has been made to agree with the quality control. Using rank of XCO_2_ individual error correlation to select bias correction parameters sometimes could miss bias correlated parameters, and hence more bias relative studies are recommend in the next stage research. The bias mostly comes from imperfect forward model and measurement (e.g. stray light and calibaration issue), and they mixed but independent for each footprint. The parameter bias correction does not only involve physical parameters but also parameters used for the spectrum correction. Therefore, we perform the bias correction separately to each footprint.

**Figure 8:**
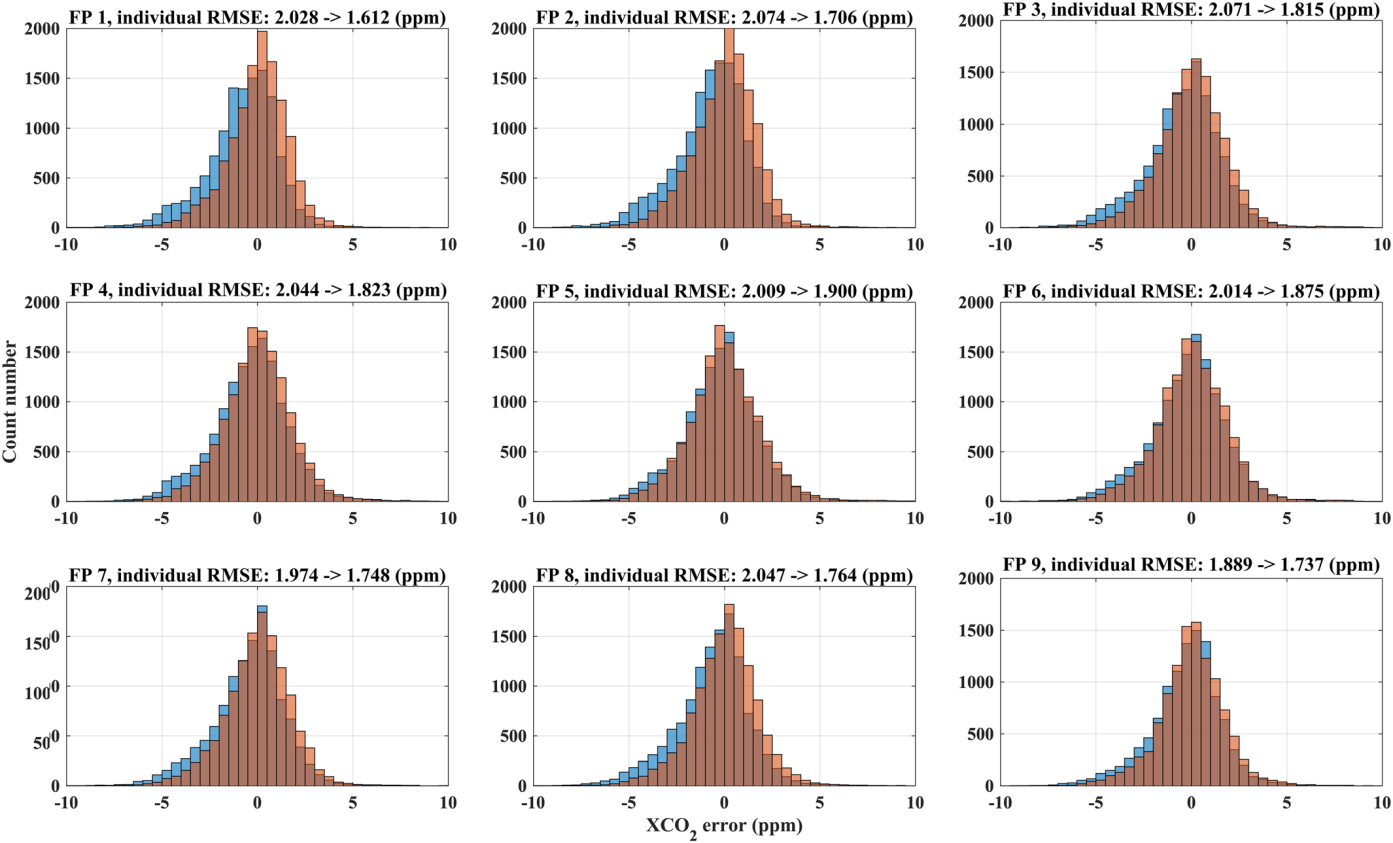
The individual XCO_2_ error (UoL‐FP/TanSat against TCCON) change with and without parameter bias correction. The orange and blue histograms indicate the XCO_2_ individual error distribution with and without bias correction. The improvement of RMSE with and without bias correction for each footprint (FP 1–9) across the swath is shown in the titles.

The effect of the bias correction is shown in Figure 8. The largest improvements in RMSE are found for footprint 1, 2, 7, and 8. Besides a parameter‐related bias, there can also be a footprint dependent bias as has been shown for OCO‐2. Typically, a stable and constant bias mainly relates to instrument effects (O'Dell et al., 2018; Wu et al., 2018). TanSat has 9 footprints in a swath across ~18 km on the ground on average. In our retrieval, we also investigate the cross‐footprint bias by subtracting the mean value of a swath when all 9 across‐track footprints are available. After the independent parameter bias correction, no obvious cross‐track footprint bias is found and the average bias is less than 0.1 ppm but with a large (>0.3 ppm) standard deviation (SD). This result is also true, when we select the swaths for low variation of surface albedo across the swath (SD < 0.01) to guarantee that the scene is comparable through all footprints (only 532 swaths satisfy this criteria). This result indicates that parameter bias correction, if carried out for each footprint independently, can reduce the across‐track bias.

## 4 XCO_2_ Retrievals Over TCCON Sites

### 4.1 The Discussions on Two‐Bands Retrieval

**Figure 9:**
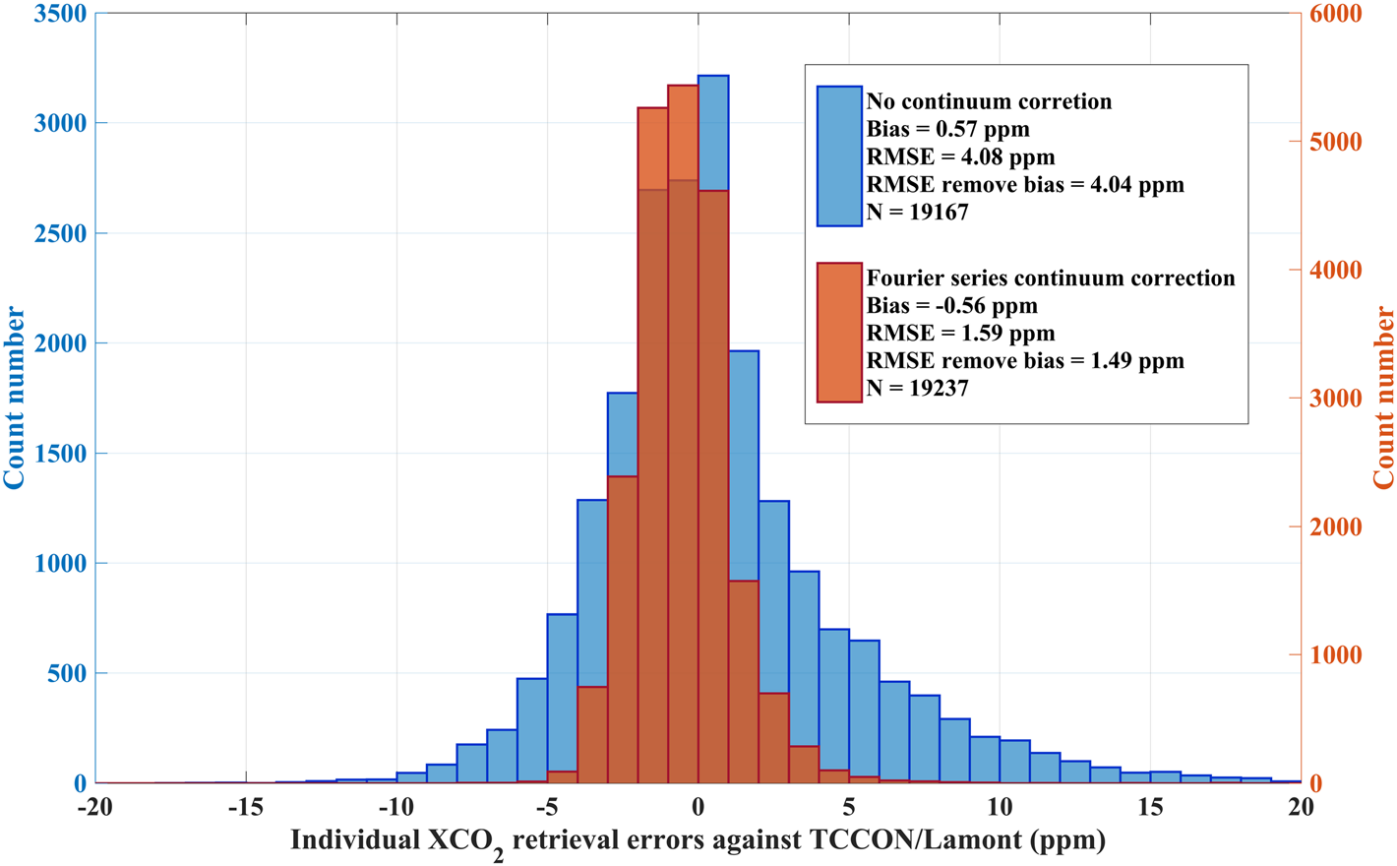
Histogram of individual XCO_2_ retrieval errors (difference between TanSat and TCCON XCO_2_) for TCCON/Lamont for clear‐sky measurements. The orange (right y‐axis) and blue (left y‐axis) give the results for the two‐band retrieval with (orange) and without (blue) Fourier series continuum correction. All data statistics in this figure passed quality control, but there is no bias correction applied. The RMSE remove bias shown in text box of this figure gives the RMSE calculated from each individual error after subtracting the overall bias.

**Figure 10:**
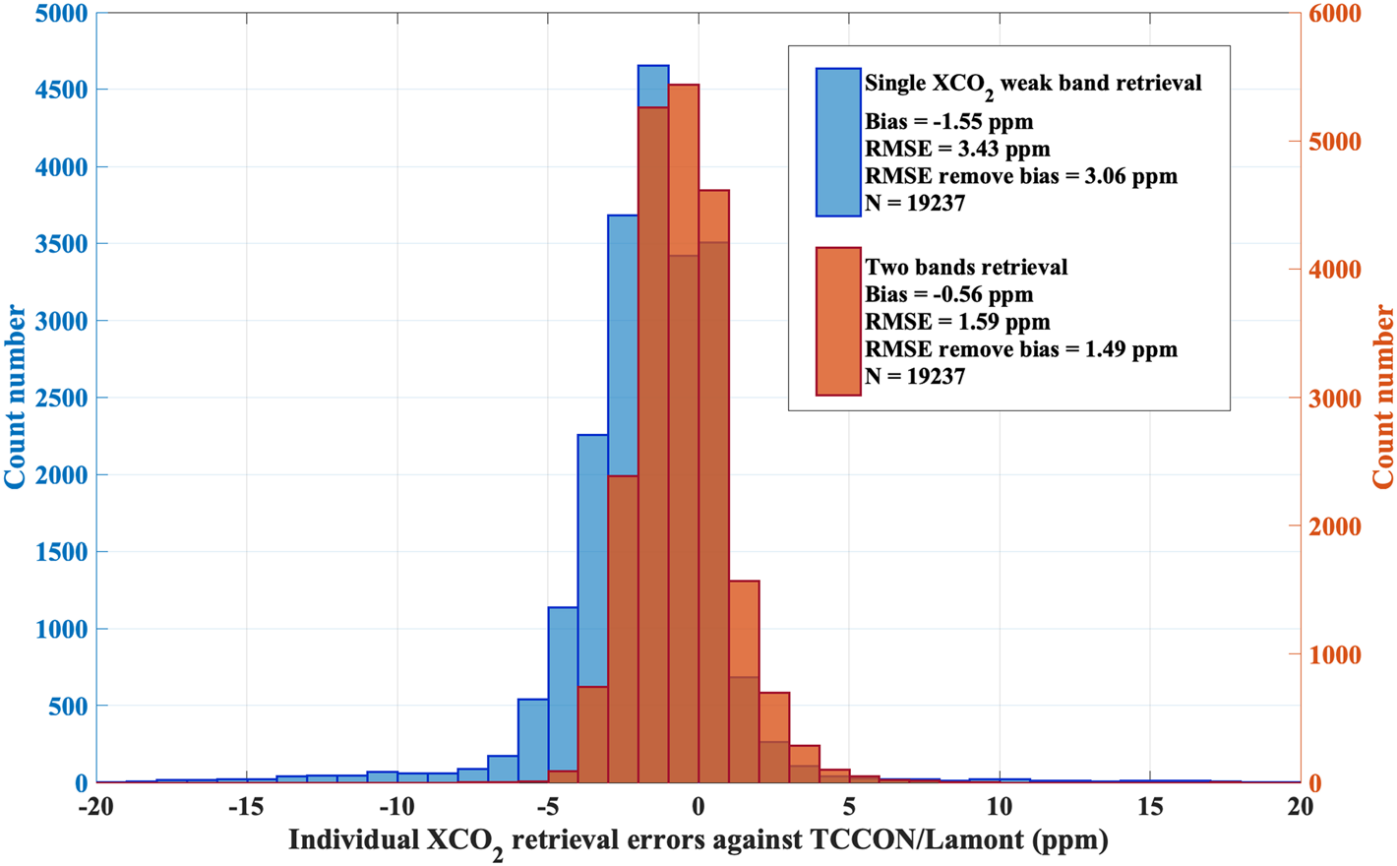
Same as Figure 9, but for a comparison between a single CO_2_ weak band retrieval (no continuum correction) and the two‐band retrieval with Fourier series continuum correction.

The Fourier series approach, introduced in section 2.3, has been instrumental in allowing a two‐bands retrieval that uses the O_2_ A band together with the weak CO_2_ band. As shown in section 2.3, the continuum correction for the O_2_ A band in the XCO_2_ retrieval leads to a significantly improved fitting residual for all 9 footprints. We have also found that the apparent surface pressure retrieval (sect.2.4) from the O_2_ A band yields reliable results when adopting the continuum correction. In this study, we retrieve XCO_2_ from TanSat nadir measurements from March 2017 to May 2018 around 20 TCCON sites (Table 2, and see detail in sect.3.1) by using the setup that has been introduced in section 2.5. The continuum correction effect (as discussed in sect.2.3) on XCO_2_ is shown in Figure 9 for TanSat retrievals around the TCCON/Lamont (USA) site using all TanSat retrievals that pass the quality control but without bias correction. The RMSE decreases from 4.08 ppm to 1.59 ppm, while the bias changes from 0.57 ppm to −0.56 ppm. We also found that more retrievals fail to converge if no continuum correction is applied, meaning that the continuum correction also improved the retrieval robustness. In summary, the continuum correction and usage of the O_2_ A band shows a significant improvement on the retrieval precision. We also compared our new approach with the TanSat XCO_2_ retrieval from a single CO_2_ band only (Figure 10). The single CO_2_ weak band retrieval has been carried out with UoL‐FP/TanSat retrieving the CO_2_ profile, surface albedo and wavelength stretch, assuming no aerosol and cloud scattering in the atmosphere. This is the same strategy used by the IAPCAS (the Institute of Atmospheric Physics Carbon dioxide retrieval Algorithm for Satellite remote sensing) retrieval to generate preliminary TanSat XCO_2_ data (Liu et al., 2018; Yang et al., 2018). The improvement is significant both on the bias and RMSE. The bias is reduced by ~1 ppm, while the RMSE decreases from 3.43 to 1.59 ppm.

### 4.2 Validation Against TCCON Measurement

**Figure 11:**
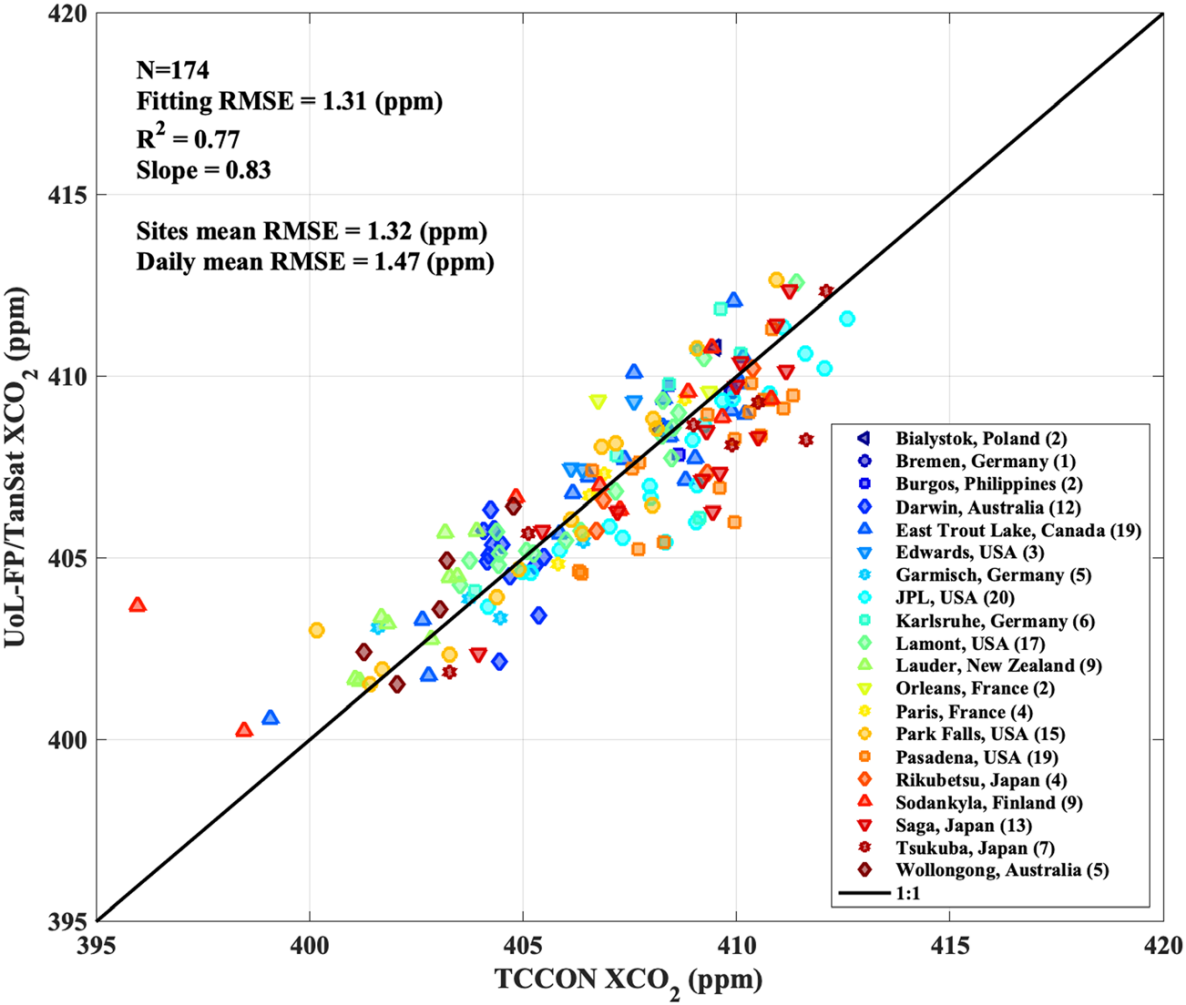
Validation of UoL‐FP/TanSat XCO_2_ retrievals against measurements from 20 TCCON sites. Each symbol represents the mean of one overpass (see detail in text section 3.1 for colocation criteria) for TanSat (only shown if the available quantity *N* > 50) and the TCCON average during the overpass (only show if the available quantity *N* > 20). The total number of overpasses per site is given in the legend. In total 174 TCCON overpasses are involved in this validation. Statistics are shown in the upper‐left corner of the figure. The daily mean RMSE is the total RMSE computed from each overpass mean and the site mean RMSE is computed by averaging the RMSE of each site. The black line indicates the 1:1 line as reference. The slope, R^2^ and fitting RMSE are the statistics from a linear regression weighted by bi‐square.

To compare TanSat retrievals to TCCON measurements, we average the quality‐controlled, bias‐corrected TanSat retrievals per overpass including all across‐track footprints for a swath, as no obvious across‐track footprint bias has been observed. Only overpasses with more than 50 soundings are used. Figure 11 shows the comparisons of the TanSat XCO_2_ average per overpass compared to the TCCON retrievals averaged over ±1 hour of the overpass time. From the 174 data pairs found, we can infer a daily mean bias of 0.08 ppm and a RSME of 1.47 ppm, which are parameters often used to characterize retrieval performance (Cogan et al., 2012; O'Dell et al., 2018; Wu et al., 2018). The linear regression has a slope of 0.83 and R^2^ of 0.77. However, these statistics can be misleading, as sites with large numbers of overpasses will have a larger weight than sites with fewer overpasses, and therefore this average is dominated by the few sites with a large number of overpasses. Consequently, the figure also displays the overall mean of the mean RMSE per site with the individual values for mean bias and RMSE given in (Table 2).

Overall, we find that mean biases are small but show a noticeable variation between sites which is partly due to the low number of data points at some sites. Considering only the seven sites with more than 10 overpasses, we find that JPL (USA), Pasadena (USA) and Saga (Japan) show a large (~1 ppm) negative bias. JPL (USA) and Pasadena (USA), for example, have a strong impact from the nearby city of Los Angles (USA), and it is suggested not to include these sites for bias correction (O'Dell et al., 2018). The linear regression with a slope of 0.83 and R^2^ of 0.77 can be improved to 0.84 and 0.92 respectively by removing measurements at Pasadena (USA), JPL (USA), Tsukuba (Japan) and Saga (Japan) (see SI section 2).

For the other four sites, Lamont, Park Falls, Darwin and East Trout Lake, we observe small biases of a few tenths of a ppm only. For Lauder (New Zealand) and Sodankyla (Finland) we also observe large biases, but the number of data points are small. In the case of Sodankyla, we find a very large outlier for one day (24 July 2017) with a large difference to TCCON (~ + 8 ppm) and to the a priori value (~ + 5 ppm). Checking cloud information from the RGB Image and MODIS cloud mask did not show cloud contamination within the satellite field of view (FOV). However, if we remove this day, the bias for this site reduces to 0.35 ppm and the RMSE to 1.25 ppm which indicates that the statistics given in Table 3 can be impacted by single outliers. It should be noted that the linear regression will be impacted by the quantity of overpasses used. In this study, we only use 15 months of TanSat nadir measurements, leading to few overpasses for many sites. Including longer time series in the future will help to increase the robustness of the results.

### 4.3 Temporal Trend and Variations

**Figure 12:**
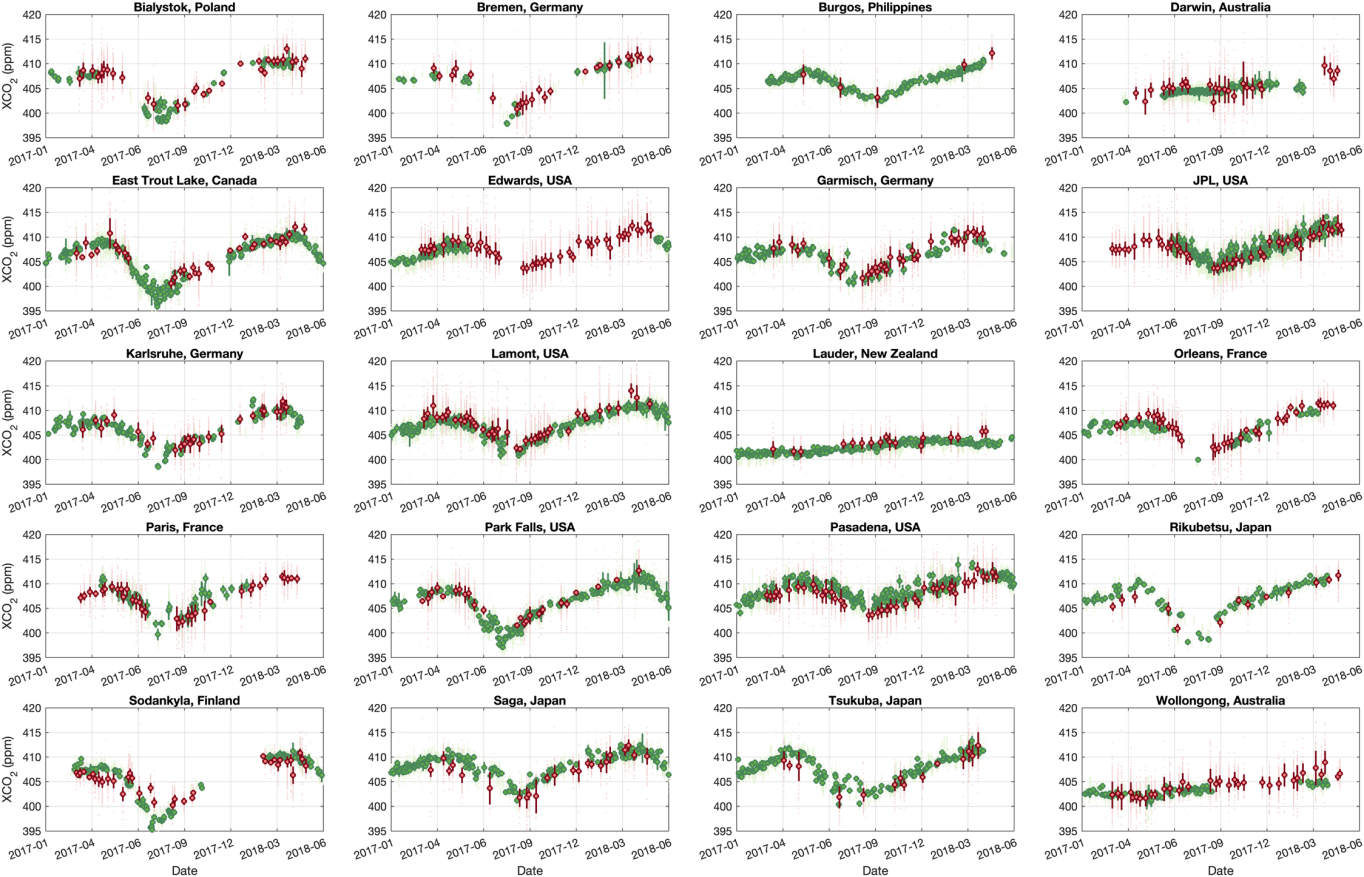
XCO_2_ time series from 2017 to 2018 for each TCCON site used in this study (site name is shown in the title of each sub‐figure). The green and red large solid circles represent TCCON and TanSat overpass mean with error bar indicating the standard deviation. The individual measurements are shown in light green and light red small points for TCCON and TanSat overpasses, respectively. The TCCON measurements are only shown when the quantity is greater than 20 and TanSat measurements are only shown when the quantity is greater than 50. This figure shows all available TCCON and TanSat data, not only TanSat‐TCCON couples, hence the data quantity shown in this figure is larger than is used for the validation.

**Figure 13:**
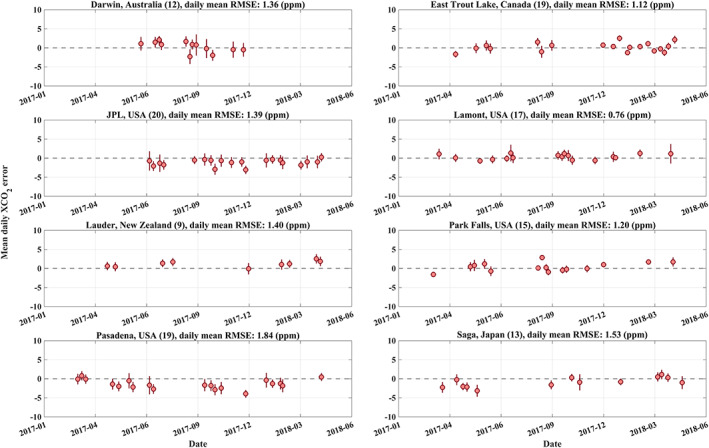
Seasonal variation of the XCO_2_ overpass mean error (difference between TanSat and TCCON XCO_2_). The data couples shown in this figure are the same as used in Figure 11. Only sites with more than 9 overpasses data points (the number of data points for each site is shown in parentheses of each sub‐figure caption) have been shown in this figure. The error bars indicate the standard deviation. The dashed gray line shows the zero‐error line as reference. The site name and RMSE are given in the title for each sub‐figure.

XCO_2_ has typically a detrended, seasonal amplitude of ~5–8 ppm from peak to trough (Lindqvist et al., 2015) in the Northern Hemisphere and roughly an annual growth of ~2 ppm globally, which is also seen from TCCON measurement from 2017 to 2018 (Figure 12). This behavior is well captured in the TanSat retrievals for both the Northern and Southern Hemisphere. For example, for Edwards (USA) the satellite measurements show a very clear seasonal variation with a peak to peak detrended variation of ~6 ppm. A + 1.36 ppm bias and RMSE of 1.39 ppm is found for this site but this is derived from 3 co‐located data‐pairs only. The negative biases found for JPL (USA) and Pasadena (USA) are clearly visible in the time series. Higher values from TanSat compared to TCCON are observed toward the end of the time series for Southern Hemisphere sites Darwin (Australia), Lauder (New Zealand) and Wollongong (Australia). A potential seasonal dependence of biases will not necessarily be corrected by the applied bias correction as it does not include parameters like solar zenith angle (or airmass) or time. To further investigate potential seasonal biases, Figure 13 shows a time series of the observed biases for sites with more than nine co‐located overpasses. Although, we find some variations of biases; for example, Saga (Japan) indicates a negative bias in the spring of 2017 and a positive bias in the spring of 2018, we do not observe a general correlation of biases with season across all sites.

## 5 Summary and Outlook

In this study, the UoL‐FP algorithm has been implemented to retrieve XCO_2_ from TanSat nadir mode observations. A major obstacle toward high‐quality retrieval of XCO_2_ from TanSat has been spectral artifacts in the fitting residual of the O_2_ A band. By analyzing the solar calibration measurement, we found that this pattern is stable with time and that it can be effectively eliminated by applying an 8‐orders Fourier series model. This correction significantly improves the fitting residual, and accordingly reduces the XCO_2_ retrieval RMSE against measurements from the TCCON site at Lamont (USA) from 4.08 to 1.59 ppm.

A data‐driven quality control and bias correction strategy has been applied to improve the data quality of the XCO_2_ retrievals. Based on the correlation of TanSat‐TCCON XCO_2_ individual error against different retrieval and forward model parameters, including Grad CO_2_, Delta Psurf, Continuum B1C3, Zeroff B2S and AlbedoB2 (see description in Table 3), a Genetic Algorithm (GA) has been used to select quality filters for 64.3% of transparency and ~2 ppm of RMSE. This leads to an overall retrieval throughput of ~18.3% (good retrievals). Compared to the manual selection of filters this achieves 13.5% more soundings for comparable RMSE. We apply a multiple linear regression for the same parameters as selected by the GA to derive a footprint dependent bias correction. After applying the bias correction, no obvious difference in remaining bias between cross‐track footprints is found.

The validation involves 20 TCCON sites with co‐location criteria within ±3° latitude/longitude and ±1 hour in time. The mean RMSE is found to be 1.47 ppm and the mean of the RMSE per site is 1.32 ppm. Typically, we find biases of a few tenths of a ppm for individual TCCON sites but larger biases (~1 ppm) are observed for some sites, especially in the proximity of major cities such as for JPL (USA), Pasadena (USA) and Tsukuba (Japan).

Previous TanSat retrievals have adopted an approach using a single (weak) CO_2_ band retrieval only. To improve this approach, the development of a 2‐band retrieval in this paper combining the O_2_ A band with the weak CO_2_ band represents a step forward in reliable TanSat retrieval as demonstrated by the improvement of the RMSE against TCCON from 3.43 to 1.59 ppm, which was found in a retrieval study of the Lamont TCCON site.

The methods used in this study, such as continuum correction, can help to improve the XCO_2_ retrieval from TanSat and subsequently the L2 data product, and hence will be applied in the IAPCAS XCO_2_ retrievals which are used for the operational processing of TanSat data. There are differences in models and retrieval strategy between the UoL‐FP/TanSat and IAPCAS/TanSat retrieval. In future studies we will investigate the impact of the differences of the two algorithms and their advantages and disadvantages. In this study we introduced an improved TanSat retrieval over land based on a two‐band retrieval. The inclusion of the strong CO_2_ band still needs to be investigated in the future and can be expected to further improve the retrieval performance by providing more information on water vapor and temperature, as well as the wavelength dependency of aerosols and clouds in the long‐wave end of the SWIR. Further studies on target mode observations would be helpful to improve the retrievals. Furthermore, the retrieval of glint mode observations over both ocean and land surfaces will need to be studied, allowing us to extend the coverage of the TanSat XCO_2_ dataset.

## Supporting information

Supporting Information S1

## Figures and Tables

**Figure A1:**
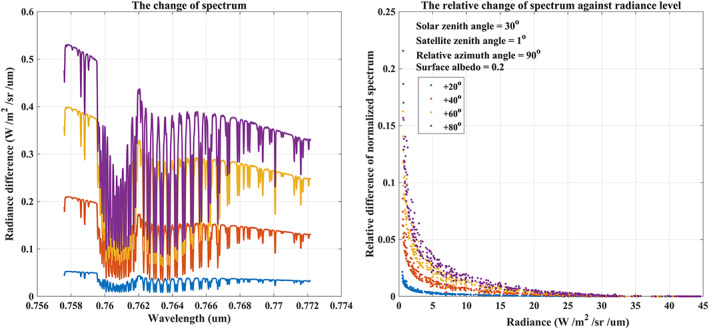
A simulation on the impact of the polarization angle on a simulated O_2_ A band spectrum. The left sub‐figure shows the changes of the spectrum when the polarization angle is 20°, 40°, 60° and 80° larger than the true angle. The color index is marked in the text box of the right sub‐figure. The right sub‐figure shows the relative change to the normalized spectrum against radiance level. Notice that the change becomes larger when the radiance decreases. This effect introduces significant errors in deep absorption lines where important information for the retrieval comes from. The simulated scene has a surface albedo of 0.2 and solar zenith angle of 30° in nadir measurement mode. The calculated polarization angle is 86.3° as calculated by the method described in section 2.1.2.

## Appendix A The Polarization Angle of TanSat Measurement

To consider polarization effects in RT and the retrieval, the polarization angle needs to be known as accurately as possible. Errors in calculated radiances that could arise from a miscalculation of the polarization angle can be as large as 20% in deep absorption lines in the O_2_ A band (Figure A1), leading to large errors in the XCO_2_ retrieval. The results shown in Figure A1 have been calculated for an aerosol‐free scene with a solar zenith angle of 30^o^ and a Lambertian albedo of 0.2 assuming the 1976 U. S standard atmosphere. However, the impact will become even worse for larger solar zenith angles; for example, Beijing, China (with latitude 40°) the SZA increases to a value larger than 60° in winter for the TanSat overpass time. The polarization impact in the CO_2_ weak and strong bands is lower in aerosol‐free scenes as the impact of Rayleigh scattering is greatly reduced, but an accurate calculation is still required in the presence of polarizing surfaces or large polarizing particles (cirrus).

Commonly, RT simulations are carried out in the local meridian plane (a plane defined by the unit vector of the local normal of a footprint and the vector from the footprint to the satellite). Unfortunately, the polarizer direction is the reference to the principal plane due to TanSat in‐flight tracking strategy (tracking the principal plane). The change of the Stokes vectors due to the rotation of the reference plane can be described by an angle defined between the principal plane and the local meridian plane (Liou, 2002; Mishchenko et al., 2004), namely the polarization angle (σ),

(A‐1) $$\cos\sigma =\frac{\cos\theta_{\mathrm{sun}}-\cos\theta_{\mathrm{obs}}\cdot \cos\Theta }{\sin\theta_{\mathrm{obs}}\cdot \sin\Theta },$$

in which *θ*_*sun*_ and *θ*_*obs*_ are the footprint solar zenith and observation zenith angles, while Θ is the scattering angle in the scattering plane, defined by the satellite line of sight and the solar direction:

(A‐2) $$\cos\theta_{\mathrm{obs}}\cos\theta_{\mathrm{sun}}+\sin\theta_{\mathrm{obs}}\sin\theta_{\mathrm{sun}}\cos\left(\Delta \varphi \right),$$

with the relative azimuth angle Δ*φ* defined by the footprint satellite azimuth and the solar azimuth angle.

In practice, due to the instrument optical design and integration strategy, the polarizer direction is parallel to (or in) the principal plane. This could lead to the light that passed the polarizer to be approximately zero (in ideal conditions) meaning that the measurement will become almost negligible when the measurement geometry meets the Brewster angle condition (solar zenith angle approaching to Brewster angle). For the purpose of avoiding this effect and increasing the received signal, the TanSat spacecraft is rotated by a + 45° yaw angle during flight. Therefore, the actual polarization angle is *σ*+45°. Then the Stokes parameter coefficients are given by,

(A‐3) a1=12,

(A‐4) a2=12cos2σ+45°,

(A‐5) a3=12sin2σ+45°,

In summary, the Stokes vectors {***I,Q,U***} at the top of atmosphere are firstly computed by the forward model and then combined to a simulation of the signal received by the instrument according to Eq. 2 in main text.

## Appendix B.

### B.1 The Genetic algorithm

Genetic algorithms (Goldberg, 1989; Holland, 1975) belong to the class of derivative‐free (Jacobi‐free) optimization methods (Rios & Sahinidis, 2013), which also includes other popular methods such as simulated annealing (Kirkpatrick et al., 1983) and particle swarm optimization (Kennedy & Eberhart, 1995). As opposed to traditional gradient‐based methods, where a cost function is minimized by updating an initial guess along a descent direction identified by computing the derivatives of the cost function with respect to its parameters (Nocedal & Wright, 1999), in derivative‐free methods the optimization is carried out through successive evaluations of the cost function itself, until a definite convergence criterion is met. An advantage of derivative‐free optimization methods compared to gradient‐based methods is that they are more capable of escaping local minima of the cost function to be optimized. Their disadvantage is that they may require a higher number of iterations before reaching the minimum. For this reason, they are particularly advantageous when the cost function is relatively inexpensive to evaluate.

Optimization in genetic algorithms is based on concepts drawn from the process of evolution taking place in biology. If by **f**(***x***) we denote a cost function to be optimized with respect to a vector x, a genetic algorithm first generates a dataset (population) {x_i_} of candidate solutions, and then randomly updates the dataset by applying two operations to its elements:

1. Mutation: one or more component of an element x are randomly perturbed
2. Cross‐over: a new element of the population is generated by combining two existing elements

All the elements in the population are then ranked with respect to a fitness function which measures the goodness of a certain element as a solution of the optimization problem, and a selection is performed by retaining a user‐defined percentage of elements which obtain the best ranking. Typically, the fitness function is the cost function itself. As a result of this selection process, a new population is created, and the random operations of mutation and cross‐over – followed by a selection – are applied to the new population. The process described above is repeated until a convergence criterion is met.

In practice, each filter there are two threshold values that need to be decided: upper boundary and lower boundary (gene). Therefore, we define two segments (upper boundaries and lower boundaries) for each individual respectively, and the combined segments as a whole, containing filters with two boundaries, and we run GA for each complexity with determined filters. The evolution is completed by multi reproductions that typically include cross‐over and mutation. Cross‐over exchanges the gene segment among individuals that win the fitness selection. The purpose of cross‐over is to combine the advantaged genes and to improve the fitness of a new generation of individuals. However, we always hope to change advantaged genes by at least a small distance toward the optimized solution. Therefore, only mutations are applied in reproduction, which has been found to be sufficient (Mandrake et al., 2013). Threshold values for each gene are initiated randomly with a quantity of 100,000 individuals ranging between the maximum and minimum values in the training dataset. For each reproduction, only 15 individuals pass the fitness selection and replace the population of the last reproduction. Therefore, the mutation is only coming from these 15 individuals that contain advantaged genes. We let the GA mutate the gene 100 times for each individual by selecting gene and according number randomly, and then 150,000 individuals have reproduced after each mutation. A convergence criterion has been chosen to stop the GA run when the RSME of the retrieval error reduces to less than 0.2% over the previous ten iterations. This criterion is looser than the one that has been introduced by Mandrake et al. (2013), but in practice, we found this works sufficiently well.
