# Supplementary material for: Toward High Precision XCO2 Retrievals From TanSat Observations: Retrieval Improvement and Validation Against TCCON Measurements
Source: J Geophys Res Atmos. 2020 Nov 9;125(22):e2020JD032794. doi: 10.1029/2020JD032794 (PMC7983077; doi:10.1029/2020JD032794)
Supplement: Supplementary file 1 — Supporting Information S1 [file JGRD-125-e2020JD032794-s001.docx]

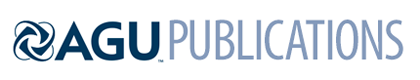


*Journal of Geophysical Research - Atmospheres*

Supporting Information for

**Toward High Precision XCO_2_ Retrievals from TanSat Observations: Retrieval Improvement and Validation against TCCON Measurements**

D. Yang^1,2,3^*, H. Boesch^1,4^, Y. Liu^2,3^, P. Somkuti^1,4,5^, Z. Cai^2^, X. Chen^2^, A. Di Noia^1,4^, C. Lin^6^, N. Lu^7^, D. Lyu^2^, R. J. Parker^1,4^, L. Tian^8^, M. Wang^3^, A. Webb^1,4^, L. Yao^2^, Z. Yin^8^, Y. Zheng^6^, N. M. Deutscher^9^, D. W. T. Griffith^9^, F. Hase^10^, R. Kivi^11^, I. Morino^12^, J. Notholt^13^, H. Ohyama^12^, D. F. Pollard^14^, K. Shiomi^15^, R. Sussmann^10^, Y. Té^16^, V. A. Velazco^9^, T. Warneke^13^, D. Wunch^17^

^1^Earth Observation Science, School of Physics and Astronomy, University of Leicester, UK. ^2^Institute of Atmospheric Physics, Chinese Academy of Sciences, China. ^3^Shanghai Advanced Research Institute, Chinese Academy of Sciences, Shanghai, China. ^4^National Centre for Earth Observation, University of Leicester, UK. ^5^Colorado State University, Fort Collins, CO, USA. ^6^Changchun Institute of Optics, Fine Mechanics and Physics, China. ^7^National Satellite Meteorological Center, China Meteorological Administration, China. ^8^Shanghai Engineering Center for Microsatellites, China. ^9^Centre for Atmospheric Chemistry, School of Earth, Atmospheric and Life Sciences, University of Wollongong, NSW, 2522, Australia. ^10^Karlsruhe Institute of Technology, IMK-IFU, Garmisch-Partenkirchen, Germany. ^11^Space and Earth Observation Centre, Finnish Meteorological Institute, Finland. ^12^National Institute for Environmental Studies (NIES), 16-2 Onogawa, Tsukuba, Ibaraki 305-8506, Japan. ^13^Institute of Environmental Physics (IUP), University of Bremen, 28334 Bremen, Germany. ^14^National Institute of Water and Atmospheric Research Ltd (NIWA), Lauder, New Zealand. ^15^Japan Aerospace Exploration Agency, Japan.^16^Laboratoire d'Etudes du Rayonnement et de la Matière en Astrophysique et Atmosphères (LERMA-IPSL), Sorbonne Université, CNRS, Observatoire de Paris, PSL Université, 75005 Paris, France. ^17^University of Toronto, Canada.

**Contents of this file**

Text S1 to S3

Figures S1 to S6

Tables S1 to S2

**Introduction**

In this Supporting Information section, two more extra studies are introduced. in Text S1, we introduce an optimized parameter bias correction method that different with the method that we used in main text. In Test S2, we introduce an attempt to remove controversial measurement and TCCON sites in validation study and discuss the improvement on result. The figures and table are correlated to the text section and marked in text.

Text S1. The optimized parameter bias correction

In this section, we tried to find an optimal combination of parameters for use in the bias correction. There are more than 141 state vector elements in our retrieval, which are shown in Table 1, with even more parameters impacting the bias; such as geometry and surface roughness. A combination that same with quality control filters (Grad CO_2_, Delta Psurf, Continuum B1C3, Zeroff B2S and AlbedoB2) has been used and the results have been described in the main text. The optimized selection will provide a better, less subjective result. In practice, we did not involve all of the one hundred plus parameters which would result in far more than a million trillion combinations. Instead, we only select 16 parameters with essential physical meaning (Table S1). Notice that those 16 parameters do not cover all 5 parameters used in the main text due to a different selection logic. The RMSE of each footprint has been used to measure the optimal results (Figure S1). We calculate the RMSE of every possible combination for each complexity, then select the combination which has the minimum RMSE as the optimal solution of the corresponding quantity. The RMSE has been found to continually decrease as more parameters are involved, but the gradient becomes very small (step decreasing RMSE < 0.01ppm) when the number of parameters is greater than 12. The validation results show a further improvement on bias and RMSE (Figure S2, S3 and S4), but the improvement is limited and a large bias from single overpass measurements still remains.

Text S2. Validation by removing anthropogenic impact risk

A large negative bias has been found in Pasadena (USA), JPL (USA), Tsukuba (Japan) and Saga (Japan) in the TCCON validation study. These sites are not recommended in the bias correction and validation study because they are highly impacted by anthropogenic activity, being very close to cities, meaning that their measurements are likely affected by these anthropogenic emissions. In this study, we show that the validation without these four sites (in Figure S5), and the statistics, improved significantly; albeit with only 1/3 of the measurements retained.


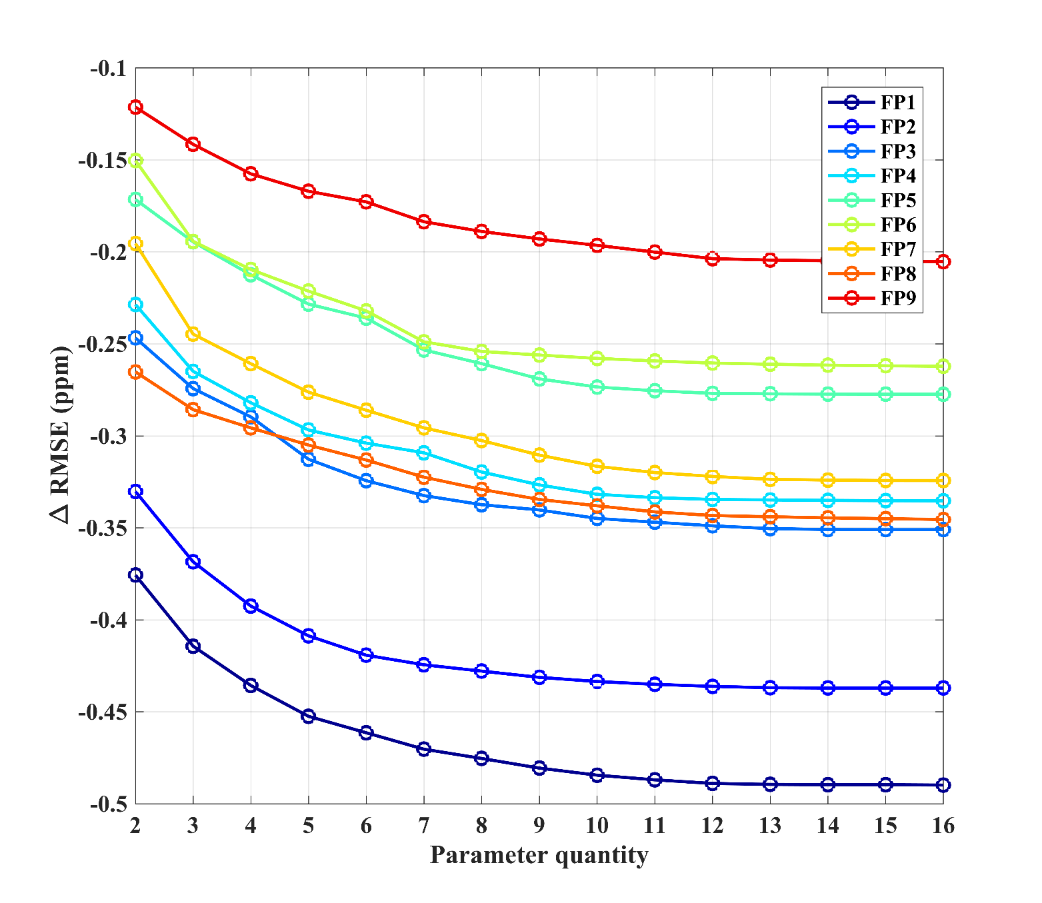


Figure S1. The RMSE difference between the retrievals with and without optimized parameter bias correction. The negative values mean the RMSE decreases when the optimized parameter bias correction is applied. Each mark on the line (9 footprint across the frame) indicates the most optimal combination of parameters from the 16 parameters dataset.


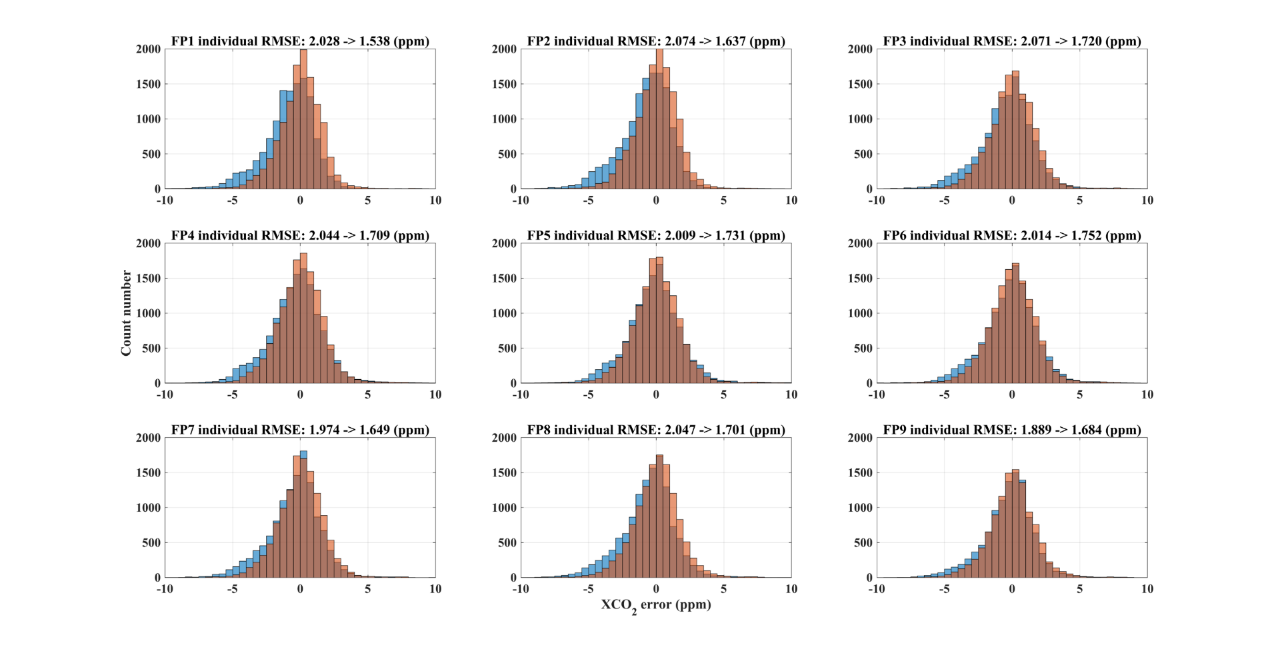


Figure S2. The performance of optimized parameter bias correction. The orange and blue histograms indicate the XCO_2_ individual error distribution with and without bias correction. The improvement of RMSE with and without bias correction for each footprint (FP 1-9) across the swath is shown in the titles.


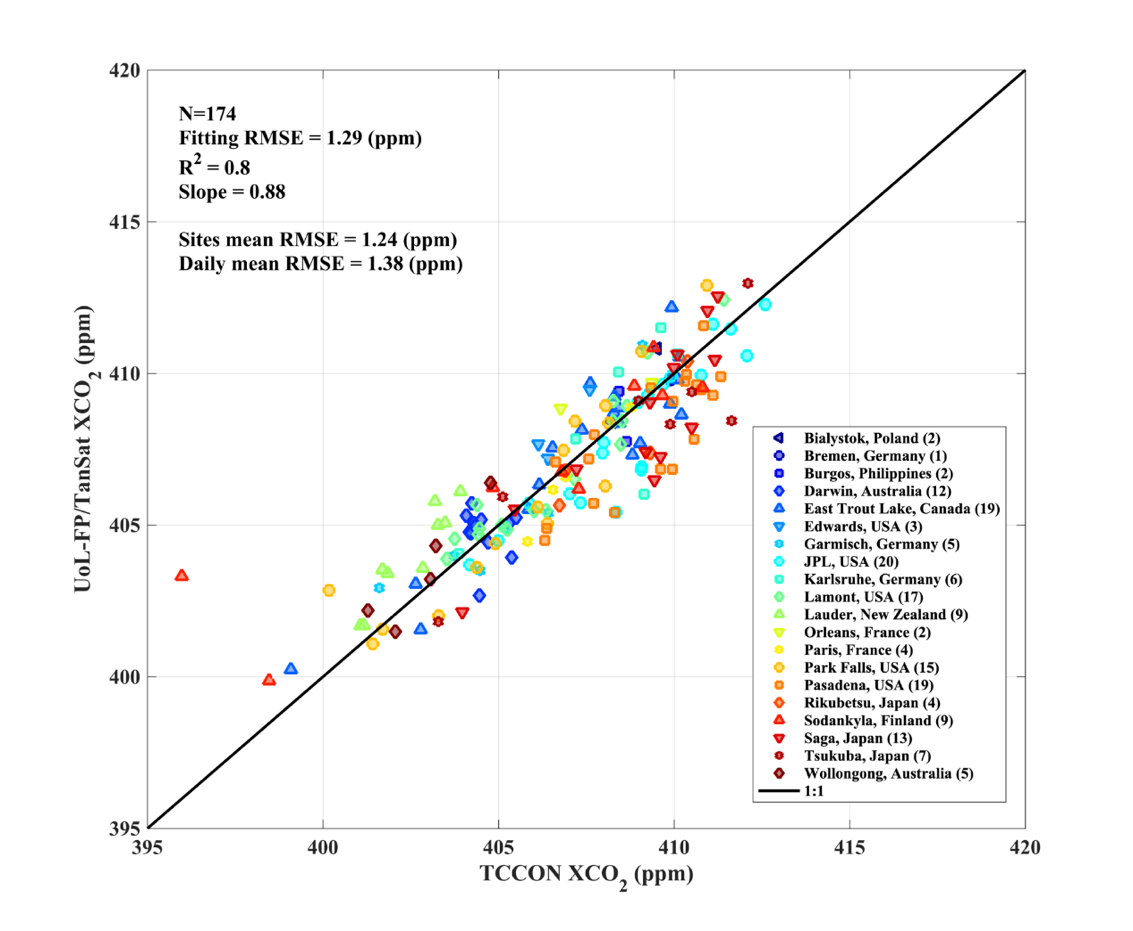


Figure S3. Validation of UoL-FP/TanSat XCO_2_ retrievals (optimized parameter bias correction) against measurements from 20 TCCON sites. Each symbol represents the mean of one overpass for TanSat (only shown if the available quantity N>50) and the TCCON average during the overpass (only show if the available quantity N>20). The total number of overpasses per site is given in the legend. In total 174 daily data couples are involved in this validation. Statistics are shown in the upper-left corner of the figure. The daily mean RMSE is the total RMSE computed from each overpass mean and the site mean RMSE is computed by averaging the RMSE of each site. The black line indicates the 1:1 line as reference. The slope, R^2^ and fitting RMSE are the statistics from a linear regression weighted by bi-square.


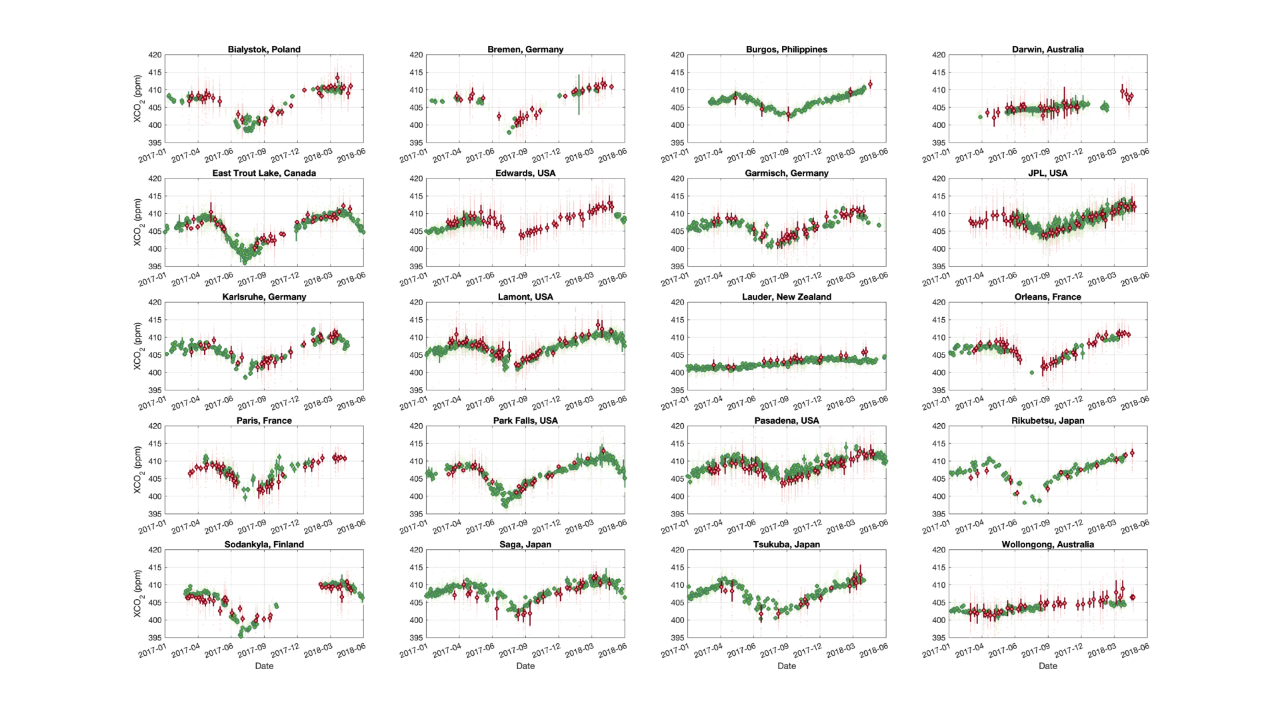


Figure S4. XCO_2_ time series from 2017 to 2018 for each TCCON site used in this study (site name is shown in the title of each sub-figure). The green and red large solid circles represent TCCON and TanSat overpass mean (with optimized parameter bias correction) with error bar indicating the standard deviation. The individual measurements are shown in light green and light red small points for TCCON and TanSat overpasses, respectively. The TCCON measurements are only shown when the quantity is greater than 20 and TanSat measurements are only shown when the quantity is greater than 50. This figure shows all available TCCON and TanSat data, not only TanSat-TCCON couples, hence the data quantity shown in this figure is larger than is used in the validation.


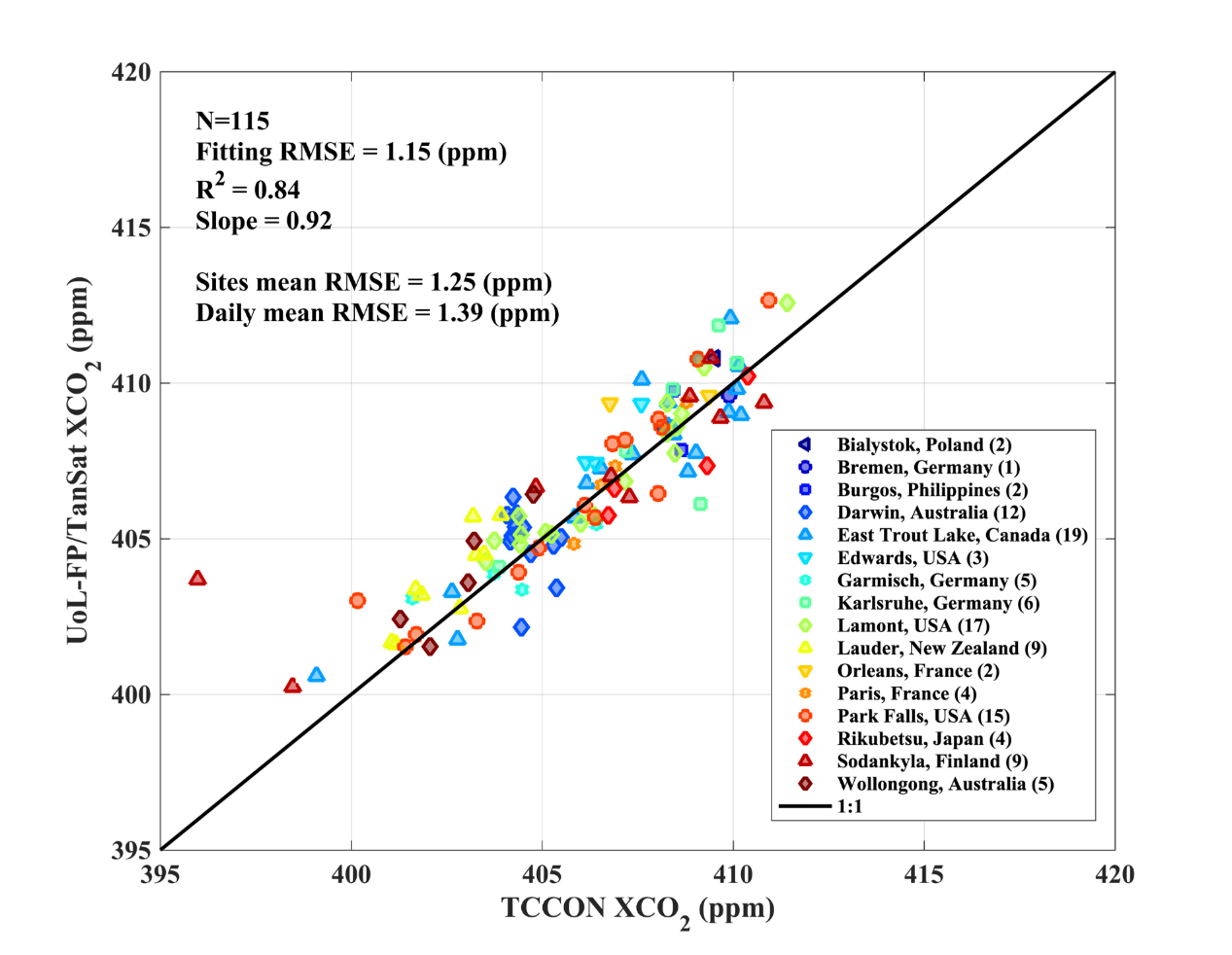
Figure S5. Same with Figure S3, but with the measurements from Pasadena (USA), JPL(USA), Tsukuba (Japan) and Saga (Japan) removed.

| Name | Description |
| --- | --- |
| Delta p_surf | The retrieval changes on surface pressure from a priori |
| AOD | Total column aerosol optical depth @ 760nm |
| OD | Total column aerosol and cloud optical depth @ 760nm |
| Zeroff B1 | Zero offset of O_2_ A band |
| Zeroff B2 | Zero offset of CO_2_ weak band |
| AlbedoB1 | Surface albedo of O_2_ A band |
| AlbedoB2 | Surface albedo of CO_2_ weak band |
| Continuum B1F | Continuum correction frequency scale of O_2_ A band |
| Continuum B2F | Continuum correction frequency scale of CO_2_ weak band |
| Overlap | Overlap of O_2_ A band and CO_2_ weak band FOV |
| RoughB1 | Surface roughness of O_2_ A band |
| RoughM | Mean surface roughness of O_2_ A band and CO_2_ weak band |
| ResidM | Mean Residual of O_2_ A band and CO_2_ weak band |
| H2Oscale | Scale factor of H_2_O |
| TempOffset | Temperature offset |
| CO2 grad | The retrieval changes of layer CO_2_ gradient between 700hPa and surface |

Table S1. The parameters list used in optimal bias correction.

Text S3. The variation of Fourier series continuum correction parameters

In our retrieval, we fit the parameters of Fourier series continuum correction. The features are not changing rapidly in time, but we cannot fix them due to there are changes between soundings. And also, the earth observation is not the exactly same as solar calibration, the incident light component is more complicate after scattered and absorbed by atmosphere. We show an example on fitting results of all parameters of footprint 5 in Figure S6, and time variation in Figure S7. The variation indicates that we still need to optimize the parameters in retrieval. In order to improve fitting in earth observation, cloud screening and XCO_2_ retrieval, we use the parameters that provide from statistics of solar calibrating fitting to be the a priori of parameters. We also list the solar calibration results and XCO_2_ retrieval of continuum correction coefficient of footprint 5 in Table S2.


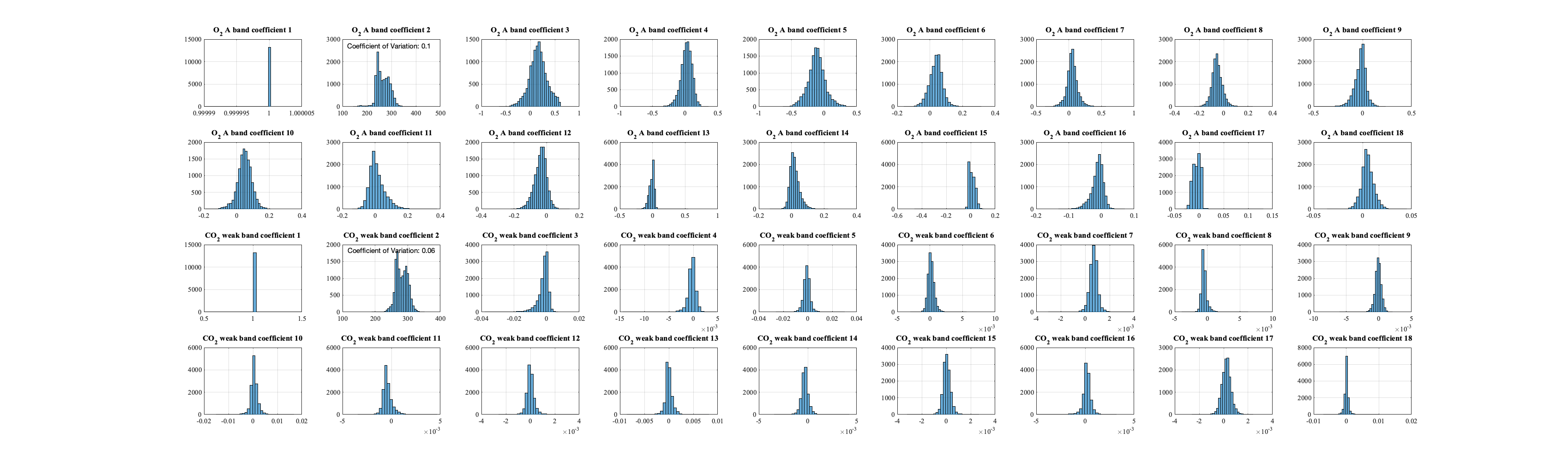


**Figure S6.** The statistics on all 36 (18 for each band) continuum correction coefficient of footprint 5. The first coefficient of each band is scale factor, so we fixed it as 1.0, the second coefficient of each band is a frequency factor in trigonometric function (ω), and then factors or each trigonometric function follows (E.q. (4) in main text). The horizontal axis is shows in each subplot title, the vertical axis is occurrence.


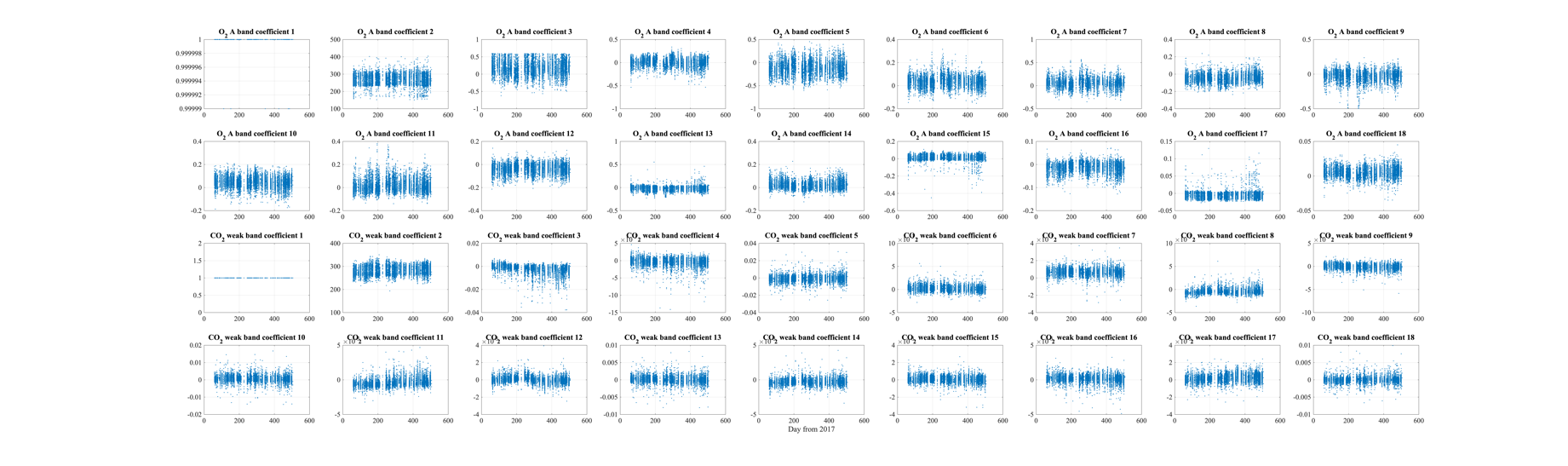


**Figure S7.** The trend of all 36 (18 for each band) continuum correction coefficient of footprint 5. The coefficient is introduced in Figure R1 caption

| coefficient | solar calibration | earth observations |
| --- | --- | --- |
| c | 1 | 1 |
| $\omega$ | 259.81 | 265.51 |
| a1 | -0.074 | 0.16 |
| b1 | -0.12 | 0.02 |
| a2 | -0.05 | -0.11 |
| b2 | -0.01 | 0.04 |
| a3 | -0.02 | 0.05 |
| b3 | 0.0036 | -0.053 |
| a4 | -0.011 | -0.024 |
| b4 | -0.0024 | 0.049 |
| a5 | -0.01 | 0.013 |
| b5 | 0.0058 | -0.038 |
| a6 | -0.0045 | -0.011 |
| b6 | 0.0098 | 0.021 |
| a7 | 0.0005 | 0.0097 |
| b7 | 0.0085 | -0.0126 |
| a8 | 0.0034 | -0.0044 |
| b8 | 0.0035 | 0.0051 |

Table S2. The mean of continuum correction coefficient in solar calibration and earth observations.
